# Congenital lipodystrophy induces severe osteosclerosis

**DOI:** 10.1371/journal.pgen.1008244

**Published:** 2019-06-24

**Authors:** Wei Zou, Nidhi Rohatgi, Jonathan R. Brestoff, Yan Zhang, Erica L. Scheller, Clarissa S. Craft, Michael D. Brodt, Nicole Migotsky, Matthew J. Silva, Charles A. Harris, Steven L. Teitelbaum

**Affiliations:** 1 Division of Anatomic and Molecular Pathology, Department of Pathology and Immunology, Washington University School of Medicine, St. Louis, MO, United States of America; 2 Division of Laboratory and Genomic Medicine, Department of Pathology and Immunology, Washington University School of Medicine, St. Louis, MO, United States of America; 3 Center for Translational Medicine, The First Affiliated Hospital of Xi’an Jiaotong University, Xi’an, Shanxi, People’s Republic of China; 4 Division of Bone and Mineral Diseases, Department of Medicine, Washington University School of Medicine, St. Louis, MO, United States of America; 5 Department of Orthopedic Surgery, Washington University School of Medicine, St. Louis, MO, United States of America; 6 Division of Endocrinology, Metabolism, and Lipid Research, Department of Medicine, Washington University School of Medicine, St. Louis, MO, United States of America; Oral Medicine, Infection and Immunity Harvard School of Dental Medicine, UNITED STATES

## Abstract

Berardinelli-Seip congenital generalized lipodystrophy is associated with increased bone mass suggesting that fat tissue regulates the skeleton. Because there is little mechanistic information regarding this issue, we generated "fat-free" (FF) mice completely lacking visible visceral, subcutaneous and brown fat. Due to robust osteoblastic activity, trabecular and cortical bone volume is markedly enhanced in these animals. FF mice, like Berardinelli-Seip patients, are diabetic but normalization of glucose tolerance and significant reduction in circulating insulin fails to alter their skeletal phenotype. Importantly, the skeletal phenotype of FF mice is completely rescued by transplantation of adipocyte precursors or white or brown fat depots, indicating that adipocyte derived products regulate bone mass. Confirming such is the case, transplantation of fat derived from adiponectin and leptin double knockout mice, unlike that obtained from their WT counterparts, fails to normalize FF bone. These observations suggest a paucity of leptin and adiponectin may contribute to the increased bone mass of Berardinelli-Seip patients.

## Author summary

Berardinelli-Seip congenital generalized lipodystrophy is a human disorder associated with increased bone mass suggesting that fat, per se, regulates the skeleton. To test this hypothesis we generated a murine model of congenital generalized lipodystrophy in which both brown and white adipose tissue are entirely depleted during embryogenesis. These “fat-free” (FF) exhibit a marked increase in bone mass throughout their body. The increased bone mass represents stimulation of bone formation and not retarded bone breakdown. Additionally, the increased bone mass of FF mice markedly increases skeletal strength and resistance to fracture. Like patients with congenital lipodystrophy, FF mice are diabetic but their metabolic state does not contribute to their increased bone mass. To identify the fat-produced molecules regulating bone mass we transplanted genetically modified adipose tissue into FF mice which established that absence of the fat-produced molecules, adiponectin and leptin, significantly enhances bone formation. These observations suggest that reducing the combined effect of adiponectin and leptin, on bone, will increase its abundance and fracture resistance.

## Introduction

The past decades have witnessed elegant studies of the relationship of energy metabolism and bone, concluding they are mutually regulatory. By this scenario, selected adipokines influence skeletal mass by directly and indirectly targeting osteoblasts and osteoclasts [1–4]. These studies, many of which provide conflicting data, typically involved pharmacologically or genetically altering adipokine abundance. The realization that different depots of white adipose tissue (WAT) have distinct physiological effects provided insight into this enigma [5–7]. While subcutaneous fat, residing predominantly in thighs and buttocks, correlates with enhanced bone mass, increased visceral fat, which characterizes the metabolic syndrome, is associated with osteoporosis [1–3, 6]. Why this distinction occurs is, however, completely unknown nor is there mechanistic proof of a cause/effect relationship between fat and abundance of bone.

We reasoned that a mouse mirroring the virtual fat depletion characterizing Berardinelli-Seip syndrome, with a robust bone phenotype, normalized by adipocyte transplantation, would be a reasonable venue to establish how "fat talks to bone". This venue would enable determination of the influence of individual fat depots as well as adipokine-modified adipocytes on the skeleton. Understanding the means by which fat, in its various forms, impacts bone, may provide a foundation for preventing and treating the skeletal complications of the metabolic syndrome. To this end, we generated mice completely deficient in WAT as well as brown adipose tissue (BAT), also postulated to enhance bone mass [8]. While other forms of murine lipodystrophy, such as the A-ZIP/F1 mouse are associated with some degree of increased skeletal mass, our fat-free (FF) mice are extremely osteosclerotic due to profound osteogenesis, despite an abundance of osteoclasts [2]. Consistent with a sympathetic contribution in both circumstances, reduction of ambient temperature partially reduces the osteosclerosis of fat-depleted mice as well as their WT counterparts. Importantly, transplantation of WT adipocyte precursors or mature white or brown fat into FF mice completely rescues their trabecular skeleton. On the other hand, transplantation of adipose tissue derived from leptin and adiponectin double deficient mice fails to do so. Thus, due at least in part to a paucity of adipose derived leptin and adiponectin, congenital absence of fat induces severe osteosclerosis by stimulating bone formation. These observations provide insight into the mechanisms of enhanced skeletal mass in human congenital generalized lipodystrophy (CGL) and suggest that reducing the combined effect of adiponectin and leptin, on bone, will increase its abundance.

## Results

### Generation of FF mice

**Fig 1:**
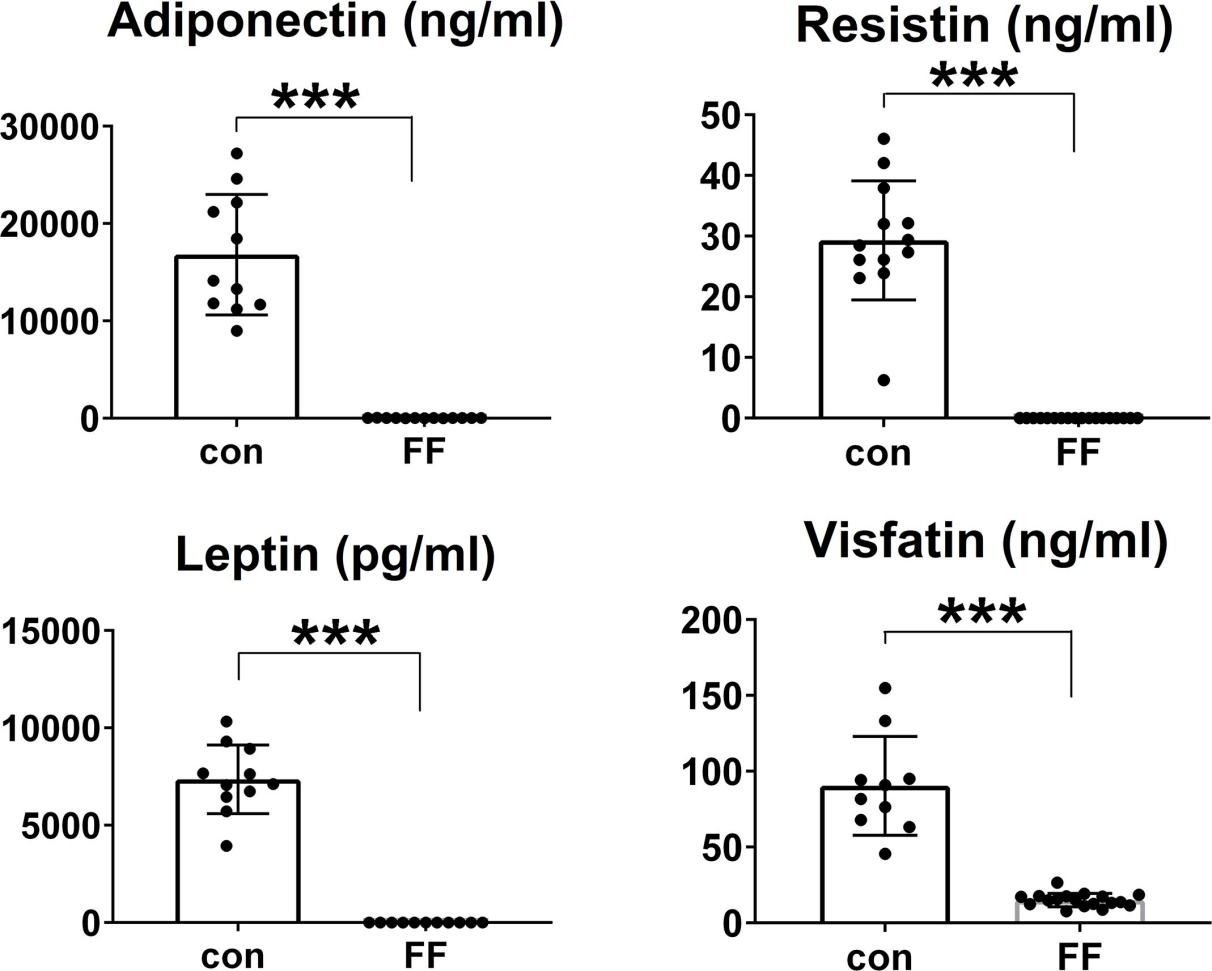
FF mice have undetectable serum adipokines. Serum adipokines of control and FF mice. Data are presented as mean ± SD. *** p<0.001 as determined by unpaired *t* test.

As described [9] generation of FF mice involved crossing those bearing a diphtheria toxin (DTA) transgene downstream of a floxed stop codon (DT-STOP^fl/fl^) to mice expressing adiponectin Cre (+/-). Cre- littermates serve as control. In keeping with the unique expression of adiponectin Cre in adipocytes and their precursors all Cre+ products of the mating contain no discernable WAT confirmed by virtually undetectable circulating adiponectin or leptin as well as visfatin and, resistin [10] (Fig 1). BAT is also absent and as a result, FF mice are cold intolerant as they require housing at 30°C to survive prior to weaning. Unexpectedly, the abundance of marrow adipocytes is unaltered in FF mice. Body weight of FF mice progressively diminishes with age, relative to WT, but the difference does not reach statistical significance until 20 weeks. There is no alteration of growth as evidenced by femoral length and FF mice have substantial splenomegaly (S1 Fig).

### Congenital absence of fat promotes osteosclerosis

**Fig 2:**
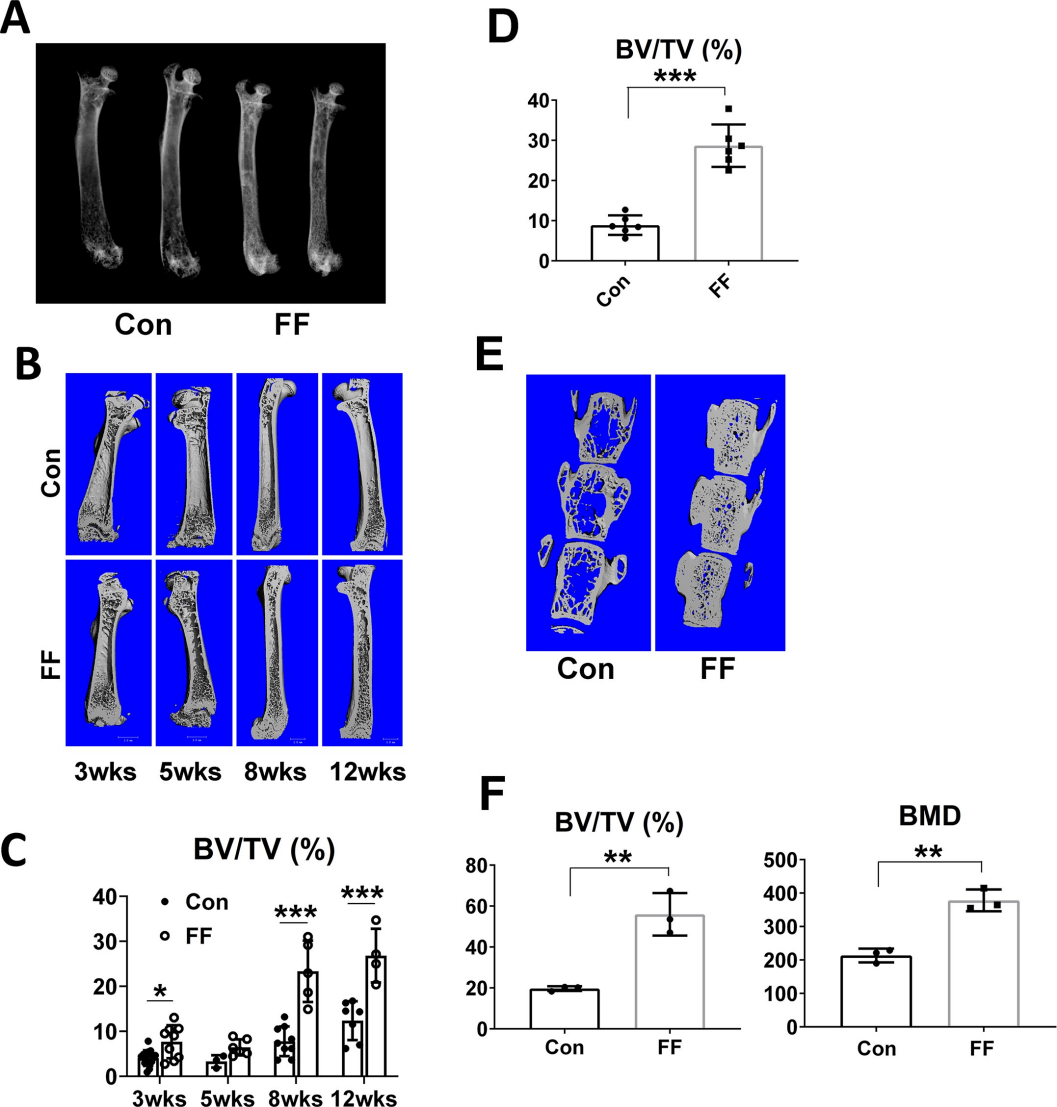
Congenital absence of fat promotes osteosclerosis. A) Radiographs of femurs of 3 month old control and FF mice. B) Age-dependent μCT images of femurs of FF and control littermates. C) Quantitative μCT analysis of B. D) Histomorphometric analysis of trabecular bone volume of 3 month old FF and control tibia. E) μCT images of vertebrae (L3-5) of 3 months old control and FF littermates. F) Quantitative μCT analysis of trabecular bone volume and bone mineral density of E. Data are presented as mean ± SD. *p<0.05; **p<0.01; *** p<0.001 as determined by unpaired t test (D, F) and 2 way ANOVA with Holm-Sidak's post hoc analysis for multiple comparisons test (C).

X-rays reveal the radiodensity of the FF skeleton is markedly increased (Fig 2A). This increase in trabecular bone mass is evident as early as 3 weeks post-partum. It maximizes at 2 months of age when BV/TV of FF mice is markedly greater than their WT littermates (Fig 2B and 2C, S2A Fig). The increase in FF trabecular bone mass is also evident histomorphometrically (Fig 2D). The osteosclerotic phenotype of fat-depleted mice is present in long bones and vertebrae (Fig 2E and 2F, S2B Fig).

Femoral cortical thickness of FF mice does not increase above control until 8 weeks and in fact, total area is reduced until that age (S3 Fig). The enhanced bone area and bone area/total area in 8 week old mice indicates that their decreased medullary area represents augmented cortical thickness due to accelerated endosteal MAR.

### Congenital absence of fat increases bone formation

**Fig 3:**
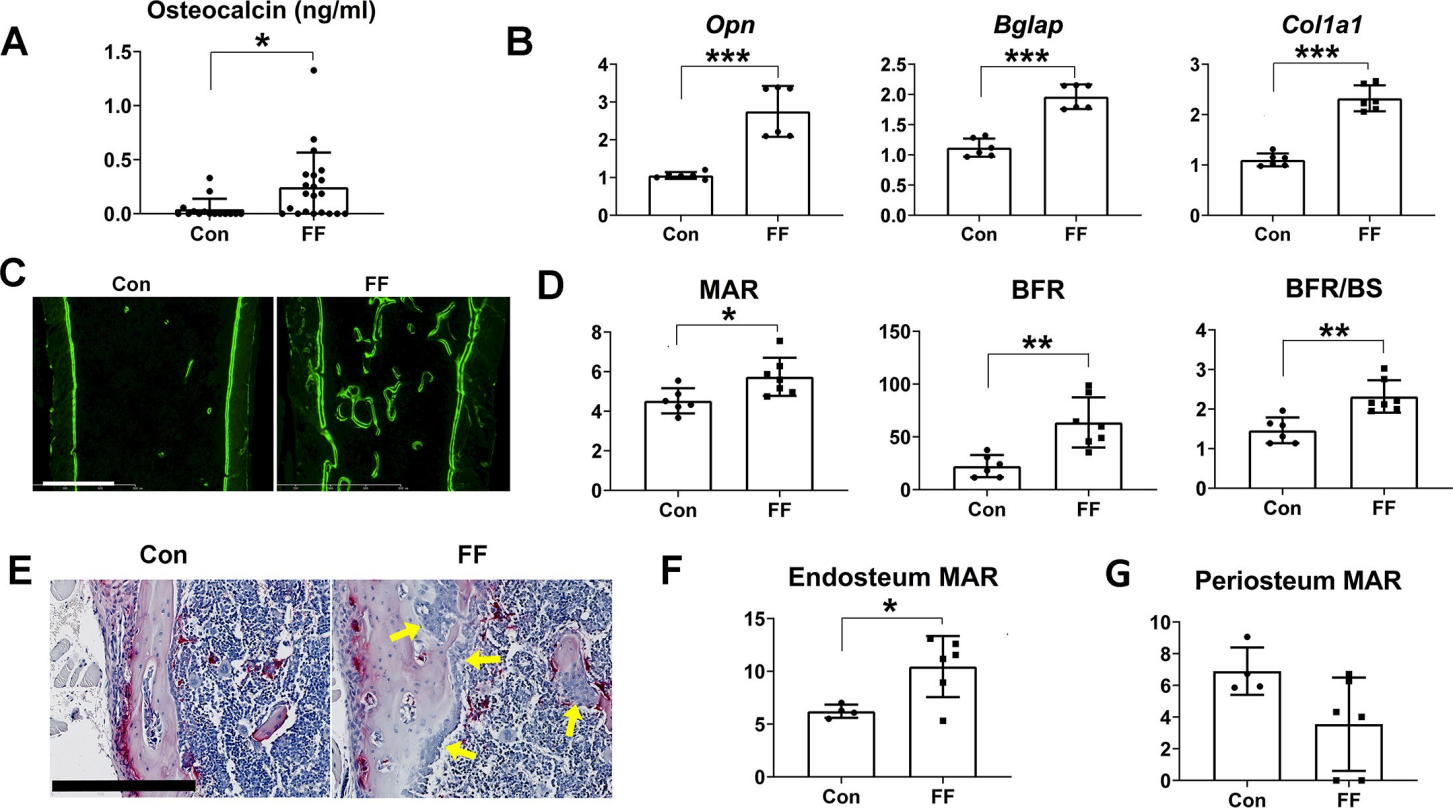
Congenital absence of fat promotes bone formation. A) Serum osteocalcin of FF and control mice. B) qPCR analysis of osteoblast differentiation markers present in RNA extracted from femurs of 6-week old FF and control mice. C) Fluorescence microscopic images of distal femur of control and FF mice administered time-spaced calcein. Scale bar: 400 μm. D) Histomorphometric analysis of total bone formation rate (BFR), bone formation rate per mm trabecular surface (BFR/BS) and mineral apposition rate (MAR) of trabecular bone. E) TRAP stained (red reaction product) control and FF femur. FF endocortical surface is lined by columnar osteoblasts (arrows) characteristic of robust bone formation. Scale bar: 800 μm. F) Histomorphometric analysis of endocortical MAR. G) Histomorphometric analysis of periosteal MAR. Data are presented as mean ± SD. *p<0.05; **p<0.01; ***p<0.001 as determined by unpaired *t* test.

Augmented bone mass may represent enhanced osteogenesis and/or decreased resorption. Increased circulating osteocalcin as well as *collagen1 α1*, *osteocalcin* and *osteopontin* mRNAs, in bone, suggests stimulated osteoblast activity contributes to the osteosclerotic phenotype of FF mice (Fig 3A and 3B). To confirm such is the case, we administered time-spaced courses of calcein. Histomorphometric analysis reveals marked acceleration of trabecular bone formation in FF mice manifest by activity of individual osteoblasts (MAR) as well when expressed in the context of trabecular surface (BFR/BS) and total bone formation rate (BFR) (Fig 3C and 3D). In keeping with increased cortical thickness, osteoblasts lining the cortical endosteal surface of FF mice assume the columnar appearance of those actively synthesizing bone (Fig 3E). Supporting this conclusion, endocortical MAR is enhanced (Fig 3F) resulting in decreased medullary area (S3B Fig). Unlike endocortical, periosteal MAR is not significantly altered in FF mice (Fig 3G).

We next determined the quantity and percentage of marrow cells expressing leptin receptor (LepR) which are reported to provide a majority of osteoblast and adipocyte progenitors [11]. Predictably, in face of markedly decreased abundance of marrow, due to its replacement by bone, the number of LepR+ cells is substantially less in FF than WT mice (S4A Fig). On the other hand, the percentage of marrow cells expressing LepR is also markedly reduced in FF mice indicating selective diminution (S4B Fig). Unexpectedly, in face of total ablation of peripheral adipose tissue, the abundance of marrow adipocytes is unaltered in FF mice (S4C Fig). These observations posit that while adiponectin Cre/DT fails to target mature marrow adipocytes in FF mice, it diminishes LepR+ precursors. Preservation of marrow adipocytes and the abundance of bone forming osteoblasts, suggest that progenitors other than those expressing LepR are functional in FF mice. This conclusion is consistent with the observation that LepR+ MSCs serve as adipocyte and osteoblast precursors only in adult mice. Alternatively, recent single cell RNA-seq based evidence establishes LepR+ cells are heterogeneous with specific subsets representing osteogenic and adipogenic precursors [12]. Thus it is possible that germ-line fat ablation selectively depletes osteogenic LepR+ subpopulations while maintaining those which are adipogenic.

### Congenital absence of fat increases osteoclast number but not function

**Fig 4:**
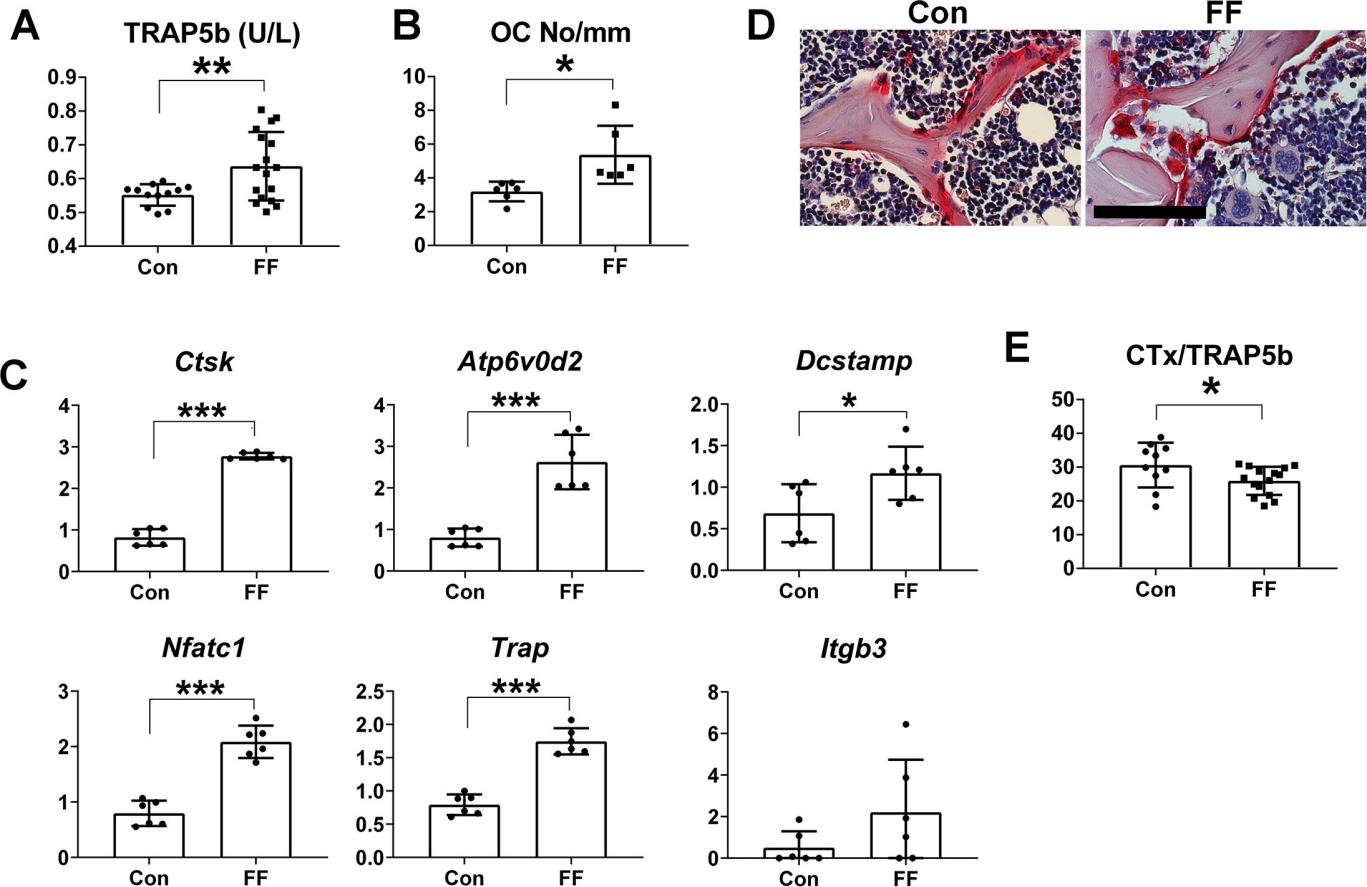
Congenital absence of fat increases osteoclast number but not function. A) Serum TRAP5b of FF and control mice. B) Histomorphometric analysis of osteoclast number/mm bone surface. C) qPCR analysis of osteoclast differentiation markers present in RNA extracted from femurs of 6-week old FF and control mice. D) TRAP stained FF and control bones. Scale Bar: 100 μm. E) Ratio of serum CTx to TRAP5b of FF and control mice. Data are presented as mean ± SD. *p<0.05; **p<0.01; ***p<0.001 as determined by unpaired *t* test.

Bone remodeling is an ever-occurring event characterized by a tethering of osteoblast and osteoclast number. Thus, in keeping with the increase in bone formation in FF mice, circulating TRAP 5b, a classical marker of osteoclast abundance, is significantly higher than that of WT counterparts (Fig 4A). The increased number of osteoclasts is confirmed by histomorphometry (Fig 4B). Osteoclast specific mRNAs are also enhanced in FF bone (Fig 4C). Many FF osteoclasts fail, however, to attach to bone and have an irregular appearance suggesting cytoskeletal and resorptive dysfunction [13] (Fig 4D). While circulating CTx of WT and FF mice are equivalent (approximately 15 ng/ml each), serum CTx, normalized to the osteoclast abundance marker, TRAP5b, is reduced confirming the resorptive activity per individual cell is decreased (Fig 4E). Thus, osteoclastic bone resorption does not contribute to the skeletal phenotype of fat-depleted mice.

### Congenital absence of fat enhances bone strength

**Fig 5:**
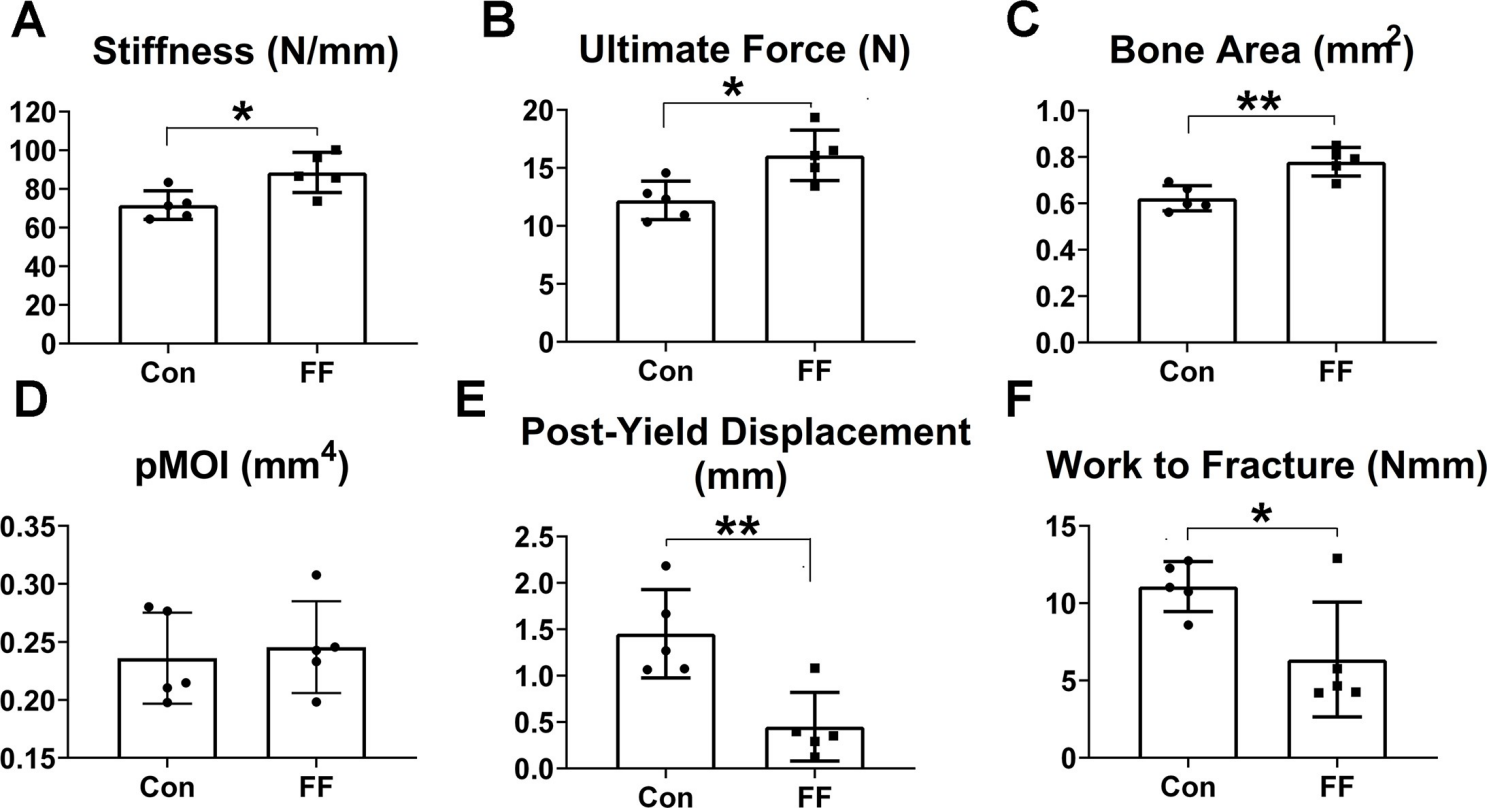
Congenital absence of fat enhances bone strength. Bending tests analysis of FF and control femoral (A) stiffness and (B) ultimate force (a measure of whole-bone strength). (C) μCT analysis of diaphyseal bone area. (D) Polar moment of inertia. (E) Post-yield displacement. (F) Work to fracture. Data are presented as mean ± SD. *p<0.05; **p<0.01 as determined by unpaired *t* test.

Increased bone mass normally amplifies the mechanical properties at the whole-bone (structural) level [14]. As such, three-point bending tests indicate that femora of 12week old FF mice are significantly stiffer (Fig 5A) with enhanced ultimate force (Fig 5B), the latter indicating superior whole-bone strength. These differences are consistent with increased bone area (Fig 5C) and cortical thickness (S3B Fig) although total area (S3B Fig) and moment of inertia (pMOI; Fig 5D) are not different. The morphological properties of FF femora reflect increased bone mass and are consistent with the enhanced endocortical but not periosteal bone formation noted above (Fig 3F and 3G). Bending tests also reveal FF femora have lower post-yield displacement (Fig 5E) and work-to-fracture (Fig 5F), indicating a more brittle phenotype. Thus, FF femora are stiffer and stronger at the whole-bone level, consistent with greater bone mass. On the other hand, FF bones have reduced post-yield displacement leading to reduced work-to-fracture and in keeping with more brittle material properties.

### Bone mass of FF and WT mice is thermogenically regulated

**Fig 6:**
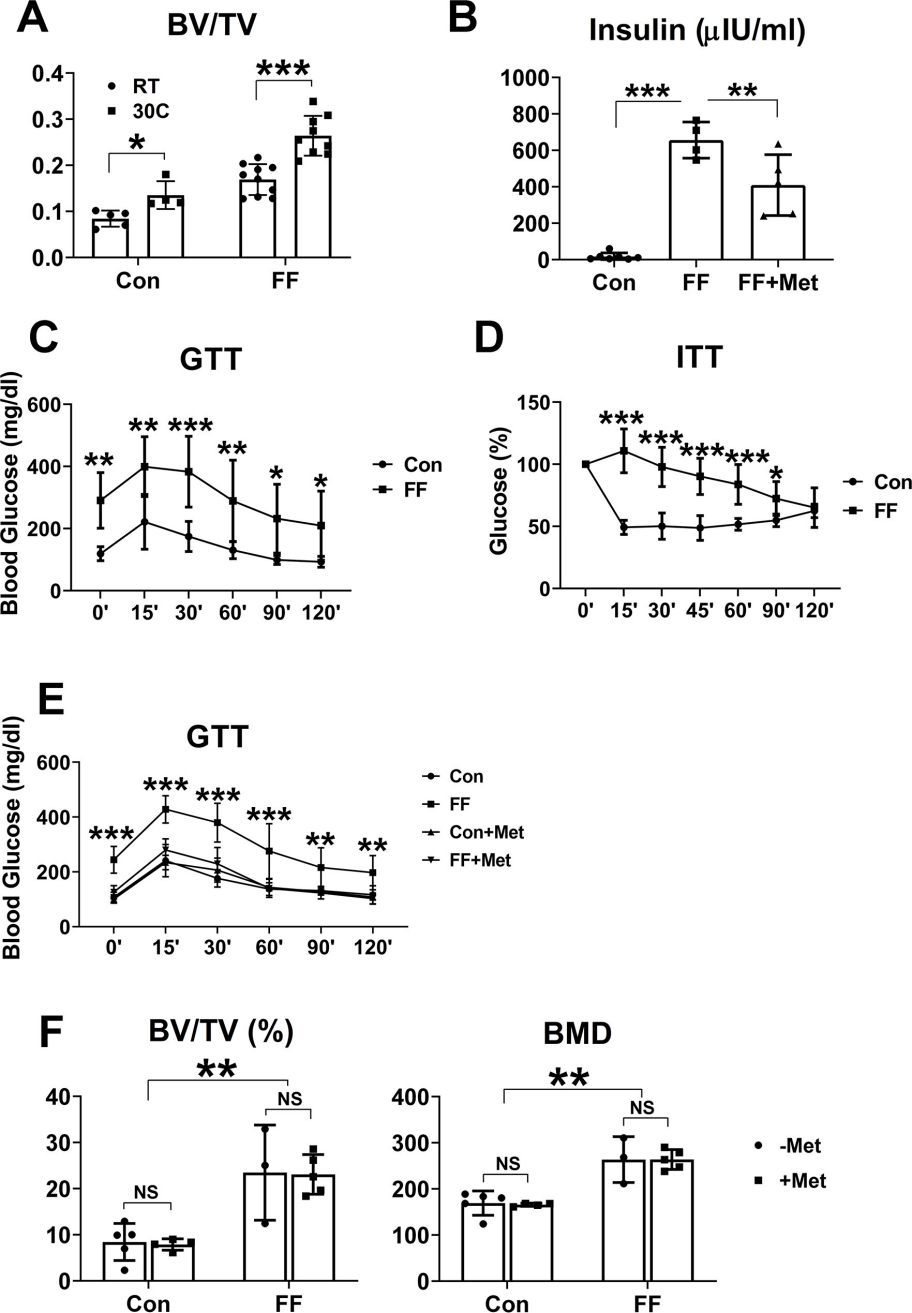
Osteosclerosis of FF mice is not caused by the metabolic syndrome. A) Femoral trabecular bone volume of control and FF mice maintained for 3 months at 23 C (RT) or 30 C. B) Serum insulin of FF mice with or without metformin treatment. Control mice serve as normal control. C) Glucose tolerance test of 2 month old FF and control littermates. D) Insulin tolerance test of 2 month old FF and control littermates. E) Glucose tolerance test of FF and control mice after 3 months treatment with or without metformin. F) μCT quantitative analysis of distal femurs of FF and control mice following 3 months with or without metformin. Data are presented as mean ± SD. *p<0.05; **p<0.01; ***p<0.001; NS: no significance as determined by 1 way ANOVA (B) and 2 way ANOVA with Holm-Sidak's post hoc analysis for multiple comparisons test (A,C,D,E,F).

While our data confirm that fat depletion induces profound osteogenesis, we have not established whether the FF environment directly and/or indirectly stimulates osteoblasts. This is particularly relevant as regards the sympathetic nervous system (SNS) as its activation may dampen osteogenesis via β-adrenergic receptors [15]. Because heat regulates skeletal mass, most likely via SNS activity, we asked if the osteosclerosis of FF mice is impacted by the temperature to which they are exposed. Thus, at weaning, FF and control littermates were maintained as usual, at 30° or at room temperature (23°) for 3 months. Consistent with sympathetic regulation, bone mass of FF mice, maintained at 23°C, is diminished relative to those kept at 30°C (Fig 6A). On the other hand, comparable ambient temperature regulation of bone mass also occurs in control mice. Given these similar environmental effects in both genotypes it is unlikely that differences in sympathetic tone account for the profound osteosclerosis of FF mice.

### Osteosclerosis of congenital generalized lipodystrophy is not caused by the metabolic syndrome

Type 2 diabetes-associated humoral factors, such as insulin, have skeletal effects. In keeping with their lipodystrophic state, FF mice are insulin resistant as documented by abnormal insulin and glucose tolerance tests (Fig 6B–6D) and severe hepatic steatosis (see below) [9]. To determine if metabolic abnormalities likely contribute to their osteosclerotic phenotype, we fed metformin, standard therapy for lipodystrophy-associated diabetes, to 3 week old FF mice, maintained at 30°C [16]. The animals were sacrificed after 3 months. While the drug normalizes glucose tolerance and significantly reduces circulating insulin it has no effect on bone mass of FF mice (Fig 6B, 6E and 6F; S5 Fig). Interestingly, unlike ovariectomized rats housed at room temperature, whose skeletal mass increases in response to metformin, that of Cre- littermates of FF mice is unaffected [17]. Thus, the osteosclerosis of FF mice likely does not meaningfully reflect their metabolic dysfunction.

### Transplanted WT adipose tissue normalizes the congenital generalized lipodystrophic skeleton

**Fig 7:**
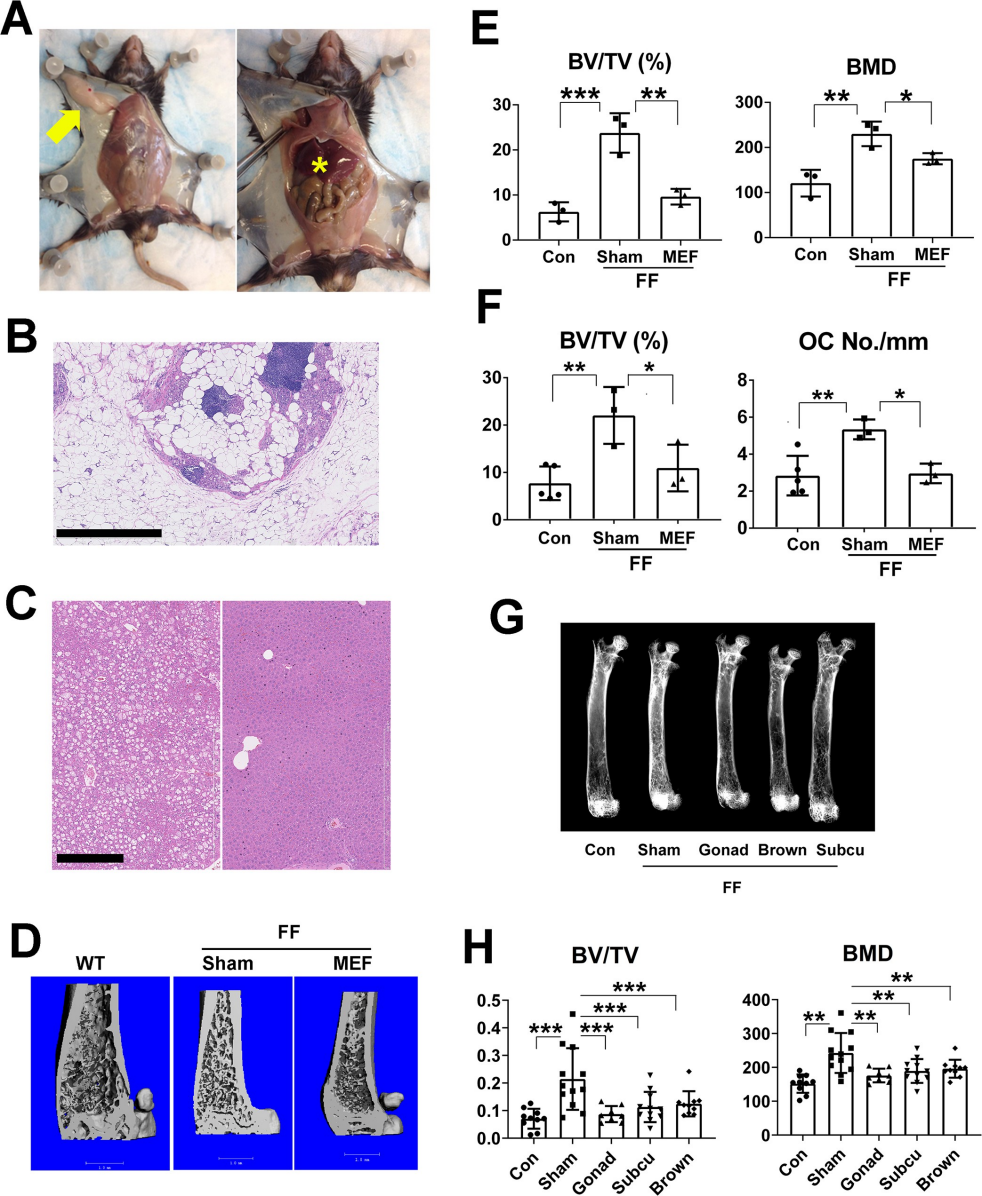
Transplanted WT adipose tissue normalizes FF skeleton. A) Left: Fat mass (arrow) in FF mouse 4 months after subcutaneous MEF transplantation. Right: WT MEF transplantation normalizes liver size of FF liver (star). B) Histological image of Fat mass in FF mouse 4 months after subcutaneous MEF transplantation; Scale bar: 1mm. C) Histological images of FF liver showing severe steatosis (left Panel) in sham operated mice and its elimination by MEF transplantation (right Panel). Scale bar: 400μm. D) μCT images and E) quantitative analysis trabecular volume and bone mineral density of distal femurs of FF mice 4 months after sham operation or MEF transplantation. F) Histomorphometric analysis of tibial trabecular bone volume and osteoclast number in WT controls and FF mice 4 months after sham operation or MEF transplantation. G) Radiographs of femurs of FF mice 3 months after sham operation or transplantation of various fat depots. H) μCT analysis of trabecular bone volume and bone mineral density of distal femurs of FF mice 3 months after sham operation or transplantation of various fat depots. Data are presented as mean ± SD. *p<0.05; **p<0.01; *** p<0.001 as determined by ANOVA with Holm-Sidak's post hoc analysis for multiple comparisons test.

We hypothesized that the skeletal effects of various fat depots and genetically modified adipocytes may be clarified by their transplantation into mice with CGL. To this end we subcutaneously transplanted 3x10^6^ mouse embryonic fibroblasts (MEFs) which differentiate into WAT, into 2 month old FF mice (Fig 7A and 7B) [18]. Within 4 months, transplantation of these adipocyte progenitors eliminates hepatic steatosis (Fig 7C) and completely reverses the trabecular osteosclerotic phenotype of the mutant mice (Fig 7D–7F; S6 Fig). To determine if the same occurs in the context of mature fat depots, we transplanted gonadal (visceral) or subcutaneous WAT or brown adipose tissue (BAT) into FF mice. Consistent with equivalent serum levels of adiponectin and leptin (S7A Fig), in each circumstance, trabecular bone mass, completely normalizes (Fig 7G and 7H, S7B and S7C Fig). Likely reflecting relative slow rate of bone remodeling, cortical thickness is unaffected after 4 months (S7D Fig). The rescue capacity of BAT likely does not reflect its effect on energy expenditure as mice with UCP1-Cre mediated BAT deletion exhibit no skeletal phenotype (S8 Fig). Thus, WT adipose tissue, regardless of origin, normalizes the FF skeleton suggesting mediation by commonly produced factors which, in the case of BAT, may reflect its “whitening” due to initial exposure to excess circulating lipids attending lipodystrophy [19] or transplanted BAT loss of sympathetic innervation.

### Absence of leptin and adiponectin moderates FF osteosclerosis

**Fig 8:**
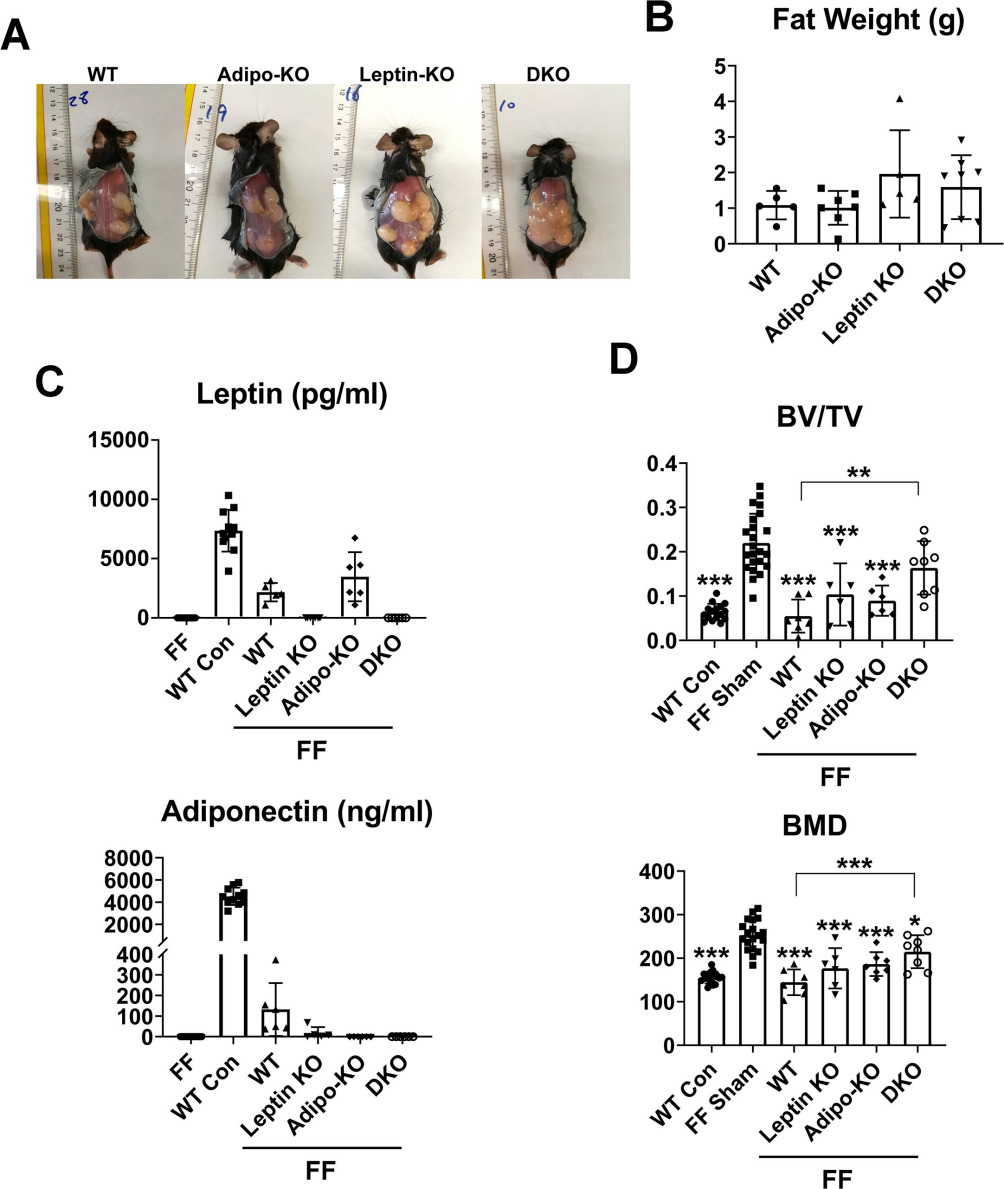
Absence of leptin and adiponectin moderates FF osteosclerosis. A) Transplanted fat depots 3 months after surgery. B) Fat depot weight 3 months after transplantation. C) Serum leptin and adiponectin of FF mice 3 months after fat depot transplantation. WT and non-transplanted FF mice serve as control. D) μCT analysis of trabecular bone volume and bone mineral density of distal femurs of FF mice 3 months after sham operation or transplantation of fat derived from WT or adipokine-deficient mice. Data are presented as mean ± SD. **p<0.01; *** p<0.001 as determined by ANOVA with Holm-Sidak's post hoc analysis for multiple comparisons test. D) comparison with FF Sham except where detailed.

Prior, albeit controversial, evidence indicates leptin and/or adiponectin exert skeletal effects [1, 2, 4]. Additionally, both adipokines are expressed by visceral, subcutaneous and brown adipocytes [20]. We therefore transplanted WT, leptin-/- (ob/ob), adiponectin-/- or double-deficient (DKO) WAT into FF mice. Three months later, the abundance of transplant-derived mutant fat was at least as great as WT (Fig 8A and 8B). Interestingly, in face of the capacity of transplanted WT WAT to completely normalize the FF skeleton, circulating leptin and particularly adiponectin, are substantially less in recipient mice than their naïve WT counterparts (Fig 8C). As expected, plasma of leptin-/- and adiponectin-/- grafted FF mice is completely devoid of their respective adipokine. While adiponectin-deficient grafts yield circulating leptin mirroring that of WT fat recipients, adiponectin is minimal in FF mice receiving leptin-/- fat. As leptin-/- fat is derived from obese ob/ob mice, the paucity of adiponectin is in keeping with the inverse relationship of the cytokine's expression and fat mass. μCT analysis established that absence of both leptin and adiponectin markedly reduces the capacity of WAT to normalize FF osteosclerosis (Fig 8D; S9A Fig). While a similar trend occurs in fat lacking either one of the cytokines, the distinction with WT fat transplantation is not significant. On the other hand, osteoclast number normalizes in FF mice regardless of adipokine expression (S9B Fig). Circulating TNFα is slightly but significantly increased in FF mice and normalized by fat transplantation (S9C Fig). Thus, absence of leptin and adiponectin, particularly when combined, contributes to the osteosclerotic phenotype of congenital generalized lipodystrophy.

## Discussion

Among the most controversial issues relating to the metabolic syndrome is the effect of obesity on bone, resolution of which will require an understanding of whether adipose tissue, *per se*, regulates skeletal biology and if so, what are the mechanisms? CGL is a rare disorder but potentially provides insights into the relationship of fat and the skeleton. For example, Berardinelli-Seip children grow rapidly and have advanced bone age. While not universal, bone mass of many CGL patients is substantially increased, a phenomenon which appears dictated by the specific causative mutation [21, 22].

We reasoned that generation of a completely fat-deficient mouse would permit use of adipose tissue transplantation to assess the effect of fat on the skeleton and most importantly, identify fat-residing bone-regulating molecules. To this end, we genetically targeted DTA to fat using adiponectin (Adipoq) Cre [7, 10]. All Cre+ mice are devoid of visible WAT and BAT. While other lipodystrophic mice, such as A-ZIP/F1, are osteosclerotic, we utilized FF mice because of their unique lack of any detectable peripheral fat depots [23]. Furthermore, for reasons unknown, marrow adipocyte abundance is unaltered in FF mice establishing that their osteosclerosis is mediated by peripheral adipose tissue, a conclusion confirmed by the complete rescue of their skeletal phenotype by gonadal, subcutaneous or brown adipose tissue. On the other hand, while FF mice mimic the phenotype of Berardinelli-Seip lipodystrophy, the pathogenesis of each differ as the human disorder is genetically based and that of the FF mouse represents adipocyte ablation [24].

Increased bone mass reflects enhanced osteoblast and/or retarded osteoclast function, an issue unresolved in lipodystrophic mouse models or patients. Bone formation is extremely robust in FF mice, a reflection of abundant osteoblasts and hyperactivity of the individual cell. This observation, taken with increased circulating osteocalcin, substantiates FF osteosclerosis is a manifestation of heightened osteogenesis.

Osteoclasts are abundant in FF mice. While circulating TNFα, a potent osteoclastogenic cytokine, is also significantly enhanced and normalized by transplanted fat, abundance of the cytokine, even in the naïve mutant mouse, is relatively low calling to question whether it contributes to increased osteoclast number [25]. While unproven, a more likely scenario holds that the abundant osteoblasts produce osteoclastogenic cytokines such as RANK ligand. Alternatively, the morphological features of cytoskeletal disorganization and diminished mobilization of CTx normalized to circulating TRAP5b, a marker of osteoclast number, indicate resorptive function of individual osteoclasts is compromised. Thus, net resorptive activity likely does not contribute to the FF bone phenotype. On the other hand, poorly resorbing osteoclasts may enhance skeletal mass by stimulating bone formation [26–28]. This event likely reflects the fact that osteoclasts promote osteogenesis not only by mobilizing matrix-residing osteoblast-activating growth factors, but also by secreting osteoblast-recruiting proteins independent of their resorptive activity. Thus, while the rate of bone formation of osteopetrotic mice, which lack osteoclasts, is suppressed, it is enhanced in those rich in dysfunctional polykaryons [28]. The same holds regarding therapy for pathological bone loss. Anti-resorptive drugs such as bisphosphonates which kill osteoclasts also suppress osteogenesis, whereas odanacatib, which impairs function of osteoclasts but does not diminish their abundance, substantially spares bone formation [29].

Among the most important contributions to understanding osteoblast biology is the discovery that their activity is sympathetically regulated and adipokines, at least in part, exert their skeletal effect by this mechanism [1, 15]. There is also evidence that maintaining mice at 23°C reduces their trabecular bone mass relative to those housed at 30°C [30]. Given that reduced ambient temperature activates the SNS, a reasonable hypothesis holds that FF mice, kept at room temperature, will have less bone than those maintained at thermoneutrality and we find such to be the case. On the other hand, the same occurs in WT mice indicating that while FF osteosclerosis may be SNS regulated, it likely occurs as a physiological event and not reflective of fat depletion.

As expected, given their lipodystrophic phenotype, FF mice are insulin resistant and steatotic. We propose these metabolic features add significance to the FF model as they replicate the complications of human CGL. Regarding a possible influence of the FF metabolic state on enhancing bone mass, evidence indicates the opposite. Specifically, fatty liver disease does not increase bone mass and in fact, promotes osteoporosis [31]. Moreover the bone mass of high fat-fed, insulin-resistant C57/BL6 mice is also decreased due to arrested bone formation [32]. Given its osteosclerotic properties and our observation that pharmacological modification of their diabetic state does not affect bone mass, it is unlikely that the metabolic syndrome of FF mice contributes substantially to their osteosclerosis and in fact may exert a negative influence superseded by robust osteogenesis.

The augmented bone mass in FF mice is contrary to the normal positive association between body size and bone mass. We recently showed in a large cohort of Large‐by‐Small advanced intercross (LG,SM AI) mice, which are WT but have a broad range of body mass due to genetic variation [33], that femur size and strength correlate positively with body and fat mass as well as serum leptin [34]. Thus, the osteosclerosis of FF mice is clearly counter to the normal physiology that links body size to bone mass. Nonetheless, biomechanical testing reveals that femora of FF mice are mechanically robust, with elevated stiffness and failure load in proportion to their increased mass. Thus, the excess bone in FF mice is mechanically competent. The only indication of possibly compromised bone properties in FF mice is decreased post-yield displacement and work-to-fracture, which reflects relatively brittle behavior of unclear origin.

We initiated this exercise to address two controversial issues relating to fat-mediated modulation of the skeleton. Both involved rescue of the CGL phenotype by fat transplantation which we first did using MEFs differentiating into adipocytes. Determining the capacity of subcutaneous and visceral WAT to alter FF osteosclerosis involved transplantation of WT individual depots into the affected mice. The fact that each depot completely normalizes FF bone suggests absence of a commonly-produced molecule(s) dampens bone formation. Moreover, reduction of bone mass by transplanted BAT challenges the commonly held position that these energy consuming adipocytes positively affect the skeleton [35]. The failure of UCP1 deletion, in adipose tissue, to impact bone mass, indicates that cure of FF osteosclerosis by BAT does not involve uncoupled energy expenditure.

A number of adipokines are proposed to impact the skeleton, most prominently, leptin and adiponectin which are virtually absent in CGL patients [36]. Despite great interest in these proteins, their skeletal properties are controversial. There is evidence that leptin diminishes bone mass by sympathetic activation via hypothalamic targeting [1, 15]. Other studies, however propose a direct effect on osteoblasts. In keeping with our observation that LepR+ marrow cells are diminished in FF mice, deletion of these cells promotes osteogenesis [37]. Alternatively, some argue that the cytokine actually increases bone mass [38–40]. The skeletal properties of adiponectin are equally complex with a majority of in vivo reports indicting osteoblast suppression while in vitro studies propose osteoblast stimulation [41]. Like leptin, both central and direct targeting of osteoblasts, by adiponectin, are postulated [4].

Transplanted WT fat completely normalizes FF bone in face of a paucity of circulating leptin and adiponectin relative to naïve WT mice. Thus, minimal expression of these adipokines may be sufficient to mediate the inhibitory effects of fat on osteogenesis and their absence likely contributes to lipodystrophy-associated osteosclerosis although lack of other adipocyte expressed factors, such as PPARϒ, may participate [2, 42, 43]. While surprising, this observation is in keeping with the capacity of small amounts of leptin to reverse the metabolic complications of lipodystrophy and the substantially greater bone mass in FF relative to other lipodystrophic mice with some residual fat [2]. On the other hand, individual deletion of either cytokine is not as effective as absence of both in maintaining the enhanced bone mass of FF mice. Whether this distinction reflects minimal expression of the other adipokine remains to be determined.

## Materials and methods

### Ethics statement

Animal work was performed according to the policies of Animal Studies Committee (ASC) at Washington University School of Medicine in St. Louis. Mice were analyzed under approved protocols and were provided appropriate care while undergoing research which complies with the standards in the Guide for the Use and Care of Laboratory Animals and the Animal Welfare Act.

### Mice

Fat Free (FF) mice were generated by mating homozygous *Lox-stop-Lox-ROSA-DTA* mice to those expressing adiponectin-Cre. BAT-deficient mice were generated by mating homozygous *Lox-stop-Lox-ROSA-DTA* mice to those expressing *UCP1-Cre*. Although no gender differences exist in phenotype, male mice were exclusively used. *Adiponectin-/*-, *leptin -/-* mice were purchased from Jackson laboratory. *Adiponectin/Leptin* double knock-out (DKO) mice were created by mating *Adiponectin-/-* and *Leptin +/-* animals.

Metformin was purchased from MP Biomedicals (Santa Ana, California) and dissolved in mouse drinking water (2 g/L) for an equivalent dose of 300 mg/kg per day, based on estimates that mice drink 1.5 mL/10 g body weight per day [44]. Fresh drinking water with Metformin was changed daily for 3 months.

### Serum ELISA assay

Blood was collected retro-orbitally under anesthesia immediately prior to sacrifice. Serum was obtained using serum separator tubes with lithium heparin (Becton Dickinson) and kept at -80C. ELISA kits were purchased from: Mouse adiponectin, leptin, adipsin, resistin and TNFα ELISA kits from R&D; Mouse Viafatin and Insulin ELISA kit from Ray biotech (Norcross, GA); Serum CTx-1 and TRAP5b kits from Nordic Bioscience; osteocalcin ELISA kit from Biomedical Technologies Inc.

### RNA extraction and quantitative qPCR

Total RNA from fresh bone was extracted using Trizol following RNA purification with RNeasy RNA purification kit and RNase free DNase digestion (Qiagen). Complementary DNA (cDNA) was synthesized from 1 μg of total RNA using the iScript cDNA synthesis kit (Bio-Rad). Quantitative qPCR was performed using the PowerUp SYBR Green Master Mix kit (Applied Biosystems) according to the kit instruction and gene specific primers. All genes amplicon length is less than 150 nucleotides. PCR reactions for each sample were performed with 7500 fast Real-Time PCR System (Applied Biosystems, Foster City, CA, USA) using the comparative threshold cycle (Ct) method for relative quantification. The glyceraldehyde-3-phosphate dehydrogenase (GAPDH) gene was used as an endogenous control. The sequences of primers are: *Itgb3*: forward: TTCGACTACGGCCAGATGATT, reverse: GGAGAAAGACAGGTCCATCAAGT; *Ctsk*: forward: AGGCAGCTAAATGCAGAGGGTACA, reverse: AGCTTGCATCGATGGACACAGAGA; *Trap*: forward: CAGCTCCCTAGAAGATGGATTCAT, reverse: GTCAGGAGTGGGAGCCATATG; *Dcstamp*: forward: ACTAGAGGAGAAGTCCTGGGAGTC, reverse: CACCCACATGTAGAGATAGGTCAG; Nfatc1: forward: CCCGTCACATTCTGGTCCAT, reverse: CAAGTAACCGTGTAGCTGCACAA; *Atp6v0d2*: forward: CAGAGCTGTACTTCAATGTGGAC; reverse: AGGTCTCACACTGCACTAGGT; *Bglap*: forward: CTGACCTCACAGATGCCAAG, reverse: GTAGCGCCGGAGTCTGTTC; *Opn*: forward: GATTTGCTTTTGCCTGTTTGG, reverse: TCAGCTGCCAGAATCAGTCACT; *Col1a1*: forward: GAGCGGAGAGTACTGGATCG, reverse: GTTAGGGCTGATGTACCAGT; *Gapdh*: forward: AGGTCGGTGTGAACGGATTTG, reverse: TGTAGACCATGTAGTTGAGGTCA.

### Flow cytometry

Bone marrow was isolated from the femur using the spin-flushing method, as previously described (Rohatgi et al., Blood Advances, 2018). Bone marrow cells from one femur/mouse were washed once in 200 μL PBS and then pelleted via centrifugation at 500 x g for 5 min at 4°C. Cells were resuspended in 50 μL of dead cell exclusion dye (ZombieUV, 1:600 in PBS, BioLegend) and incubated on ice for 10 min covered in foil. The ZombieUV stain was quenched with 200 μL FACS Buffer (PBS containing 2.5% heat-inactivated FBS and 2.5 mM EDTA), and cells were collected via centrifugation, as above. Cells were resuspended in 50 μL FcBlock (10 μg/mL of rat anti-mouse CD16/32 antibody, clone 2.4G2, BD Biosciences) and incubated on ice for 10 min covered in foil. An equal volume (50 μL) of 2X concentrate of primary antibody cocktail was added for a final staining volume of 100 μL. The primary antibody cocktail contained rat anti-mouse CD45-BUV395 (BD Horizon, clone 30-F11, final dilution factor 1:200), rat anti-mouse TER-119-APC (BioLegend, clone TER-119, 1:200), rat anti-mouse CD41-BV421 (BioLegend, clone MWReg30, 1:300), rat anti-mouse/human CD11b (BioLegend, clone M1/70, 1:400), and rat-anti mouse Leptin receptor (LepR)-biotin (R&D Systems, polyclonal, 1:50) in Brilliant Stain Buffer (BD Biosciences) containing 10 μg/mL FcBlock. Cells were stained for 30 min on ice and then washed 3 times in 200 μL FACS Buffer. The cells were resuspended in 100 μL of streptavidin-PE/Cy7 (BioLegend) and incubated on ice for 20 min before being washed 3 times in 200 μL FACS Buffer. Cells were resuspended in 200 μL FACS Buffer, and CountBright Absolute Counting Beads (Molecular Probes, 25 μL) were added to quantify total cell numbers using lot-specific bead concentration of 51,000 beads per 50 μL (Lot number 2014181). Flow cytometry data were analyzed with FlowJo version 10 (Becton, Dickinson & Company). LepR+ stromal cells were defined as singlet, live, CD45^–^, TER-119^–^, CD41^–^, CD11b^–^, LepR^+^ cells.

### GTT and ITT

Glucose tolerance tests (GTT) were performed on 2 month old male mice in clean cages subjected to starvation with free access to water for 6 hours. Mice were weighed and a small amount of blood was obtained from tail vein for baseline (time 0) glucose measurement. Mice were then injected intraperitoneally with 50% sterile dextrose (1 mg/g body weight). Tail blood glucose was determined at 15, 30, 60, 90 and 120 min after challenge using a Bayer Contour glucometer. For insulin tolerance test (ITT), 2 month old male mice were placed in clean cages without food and free access to water. Following a 6 hr fast, the mice were weighed and baseline glucose reading was taken using Bayer Contour glucometer. Mice were injected intraperitoneally with human insulin (Humulin, Eli-Lilly) at a dose of 0.7U/Kg body weight and blood glucose measured at 15, 30, 45, 60, 90, 120 min after insulin injection.

### Histology and histomorphometry

Femur and Tibia were fixed in 10% neutral buffered formalin, followed by decalcification in 14% EDTA for 10 days, paraffin embedding, and TRAP staining. Static and dynamic histomorphometric parameters were measured using BioQuant OsteoII (BioQuant Image Analysis Corporation, Nashville, TN) in a blinded fashion.

### Calcein labeling

2 month old male FF and control littermates were injected intraperitoneally with calcein (Sigma) (7.5 mg/kg of body weight) on days 6 and 2 before sacrifice. Non-decalcified histological sections of femur were analyzed using BioQuant OsteoII (BioQuant Image Analysis Corporation, Nashville, TN).

### Microcomputed tomography (μCT)

Trabecular bone was scanned using μCT40 scanner (Scanco Medical AG, Bassersdorf, Switzerland; 55 kVp, 145 μA, 300 ms integration time, 16 μm voxel size). A lower threshold of 250 was used for evaluation of all scans. The trabecular bone region of interest consisted of 100 slices that were drawn starting with the first slice in which condyles and primary spongiosa were no longer visible, constituting 1.6 mm in length. The region of interest of cortical analyses consisted of 50 slices covering a length of 0.8 mm at the femur midshaft.

### Fat transplantation

Primary mouse embryonic fibroblasts (MEFs) were prepared from WT C57/BL6 E14 embryos as described [9, 45]. MEFs were injected subcutaneously at the sternum of 2 month old FF mice as reported [9, 46]. Mice were sacrificed 4 months after transplantation.

Mature fat depots were transplanted as described [47] with slight modification. 2 month old FF mice were anesthetized with isoflurane. Donor fat pads from 6–8 week old WT or adipokine deficient mice were placed into sterile PBS and cut into 100-150mg pieces. The grafts were implanted subcutaneously through small incisions in the shaved skin of the back, with 1 piece per incisions. 6 pieces of fat graft were implanted into each FF mouse. After surgery the mice were housed individually for a week and then 5 mice per cage. Mice were sacrificed 3 months after transplantation.

### Biomechanics

Femora (n = 5 per group) were scanned by microCT at the midshaft (Scanco uCT40; 70 kVp, 114 mA, 300 ms integration time, 10 um voxel size, 100 slices) to determine cross-sectional geometric properties. They were then mechanically tested to failure in three-point pending (Instron 8841; support span: 7 mm; displacement rate: 0.1 mm/sec). Failure occurred directly beneath the loading point, at the 50% length of the femur. Force-displacement data were collected and analyzed to determine whole-bone (structural) mechanical properties (stiffness, ultimate force, post-yield displacement, work-to-fracture) [14].

### Statistics

Statistical significance was determined using Student’s *t* test, one way or 2 way ANOVA test with Holm-Sidak post–hoc test with adjustment for multiple testing. Data are expressed as mean ± SD. *p < 0.05, **p < 0.01, ***p < 0.001 in all experiments.

## Supporting information

S1 Fig FF mice have splenomegaly. Age-dependent a) Body weight; B) femur length; C) ratio of spleen per body weight of FF and control littermates. Data are presented as mean ± SD.**p<0.01; *** p<0.001 as determined by unpaired *t* test (C) and 2 way ANOVA with Holm-Sidak's post hoc analysis for multiple comparisons test (A,B).
(TIF)

S2 Fig Congenital absence of fat promotes osteosclerosis. μCT quantitation of structural model index (SMI), trabecular number (Tb.N), trabecular thickness (Tb.Th), trabecular spacing (Tb.Sp) and connection density (Conn.Dens) of 3 month old control and FF. A) femur and B) vertebrae. Data are presented as mean ± SD. *p<0.05; ***p<0.001 as determined by unpaired *t* test.
(TIF)

S3 Fig Congenital absence of fat promotes osteosclerosis. A) Age-dependent μCT images of femur diaphyseal mid-shaft region of FF and control littermates; B) μCT quantitation of A. Data are presented as mean ± SD. *p<0.05; **p<0.01; *** p<0.001 as determined by 2 way ANOVA with Holm-Sidak's post hoc analysis for multiple comparisons test.
(TIF)

S4 Fig Congenital absence of fat decreases marrow LepR+ cells. A) Flow cytometry assay of Lin-LepR+ cell number B) and ratio of Lin-Lep+ per total cell in femur marrow of 7 weeks old FF and control littermates. Data are presented as mean ± SD. *p<0.05; **p<0.01; as determined by unpaired *t* test. C) Histological section of 3 month old FF and control tibia stained for TRAP activity (red reaction product). Marrow adipocytes are present in both genotypes (arrow).
(TIF)

S5 Fig Osteosclerosis of FF mice is not caused by the metabolic syndrome. μCT quantitative analysis of distal femurs of FF mice following 3 months with or without metformin. Data are presented as mean ± SD.
(TIF)

S6 Fig MEF transplantation normalizes FF skeleton. μCT quantitative analysis of distal femurs of FF mice 4 months after sham operation or MEF transplantation. Data are presented as mean ± SD. *p<0.05; *** p<0.001 as determined by ANOVA with Holm-Sidak's post hoc analysis for multiple comparisons test.
(TIF)

S7 Fig Transplanted WT adipose tissue normalizes FF skeleton. A) Serum leptin and adiponectin of FF mice 3 months after WT fat depot transplantation. μCT B) images and C) quantitative analysis of distal femurs of FF mice 3 months after sham operation or transplantation of various fat depots. D) μCT quantitative analysis of femur diaphyseal mid-shaft region of FF mice 3 months after sham operation or transplantation of various fat depots. Data are presented as mean ± SD. *p<0.05; **p<0.01; *** p<0.001; NS, not significant as determined by ANOVA with Holm-Sidak's post hoc analysis for multiple comparisons test.
(TIF)

S8 Fig BAT deletion does not increase bone mass. μCT quantitative analysis of femurs of three month old DTA-UCP1 Cre mice. Data are presented as mean ± SD.
(TIF)

S9 Fig Absence of leptin and adiponectin moderates FF osteosclerosis. A) μCT analysis of distal femurs of FF mice 3 months after sham operation or transplantation of fat derived from WT or adipokine-deficient mice; B) Histomorphometric analysis of osteoclast number of FF and control femur 3 months after sham operation or transplantation of fat derived from WT or adipokine-deficient mice; C) Serum TNFα of control mice and FF mice 3 months after sham operation or transplantation of fat derived from WT or adipokine-deficient mice. Data are presented as mean ± SD. *p<0.05; **p<0.01; *** p<0.001 as determined by ANOVA with Holm-Sidak's post hoc analysis for multiple comparisons test. A) Comparison with Sham except where detailed.
(TIF)
