# Supplementary figures and images for: Congenital lipodystrophy induces severe osteosclerosis
Source: PLoS Genet. 2019 Jun 24;15(6):e1008244. doi: 10.1371/journal.pgen.1008244 (PMC6611650; doi:10.1371/journal.pgen.1008244)

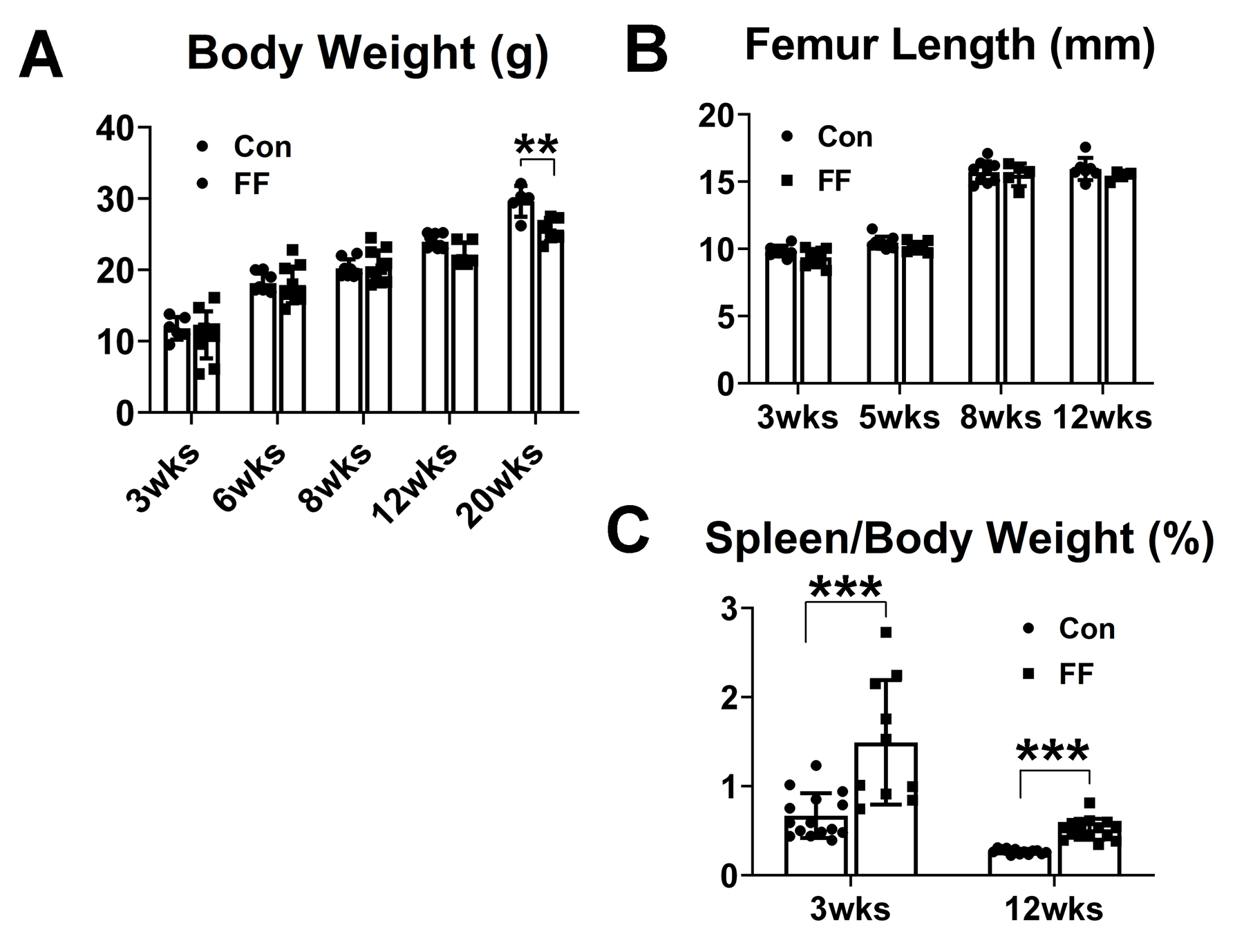

Supplement: S1 Fig — Age-dependent a) Body weight; B) femur length; C) ratio of spleen per body weight of FF and control littermates. Data are presented as mean ± SD.**p<0.01; *** p<0.001 as determined by unpaired t test (C) and 2 way ANOVA with Holm-Sidak's post hoc analysis for multiple comparisons test (A,B). (TIF) [file pgen.1008244.s001.tif]

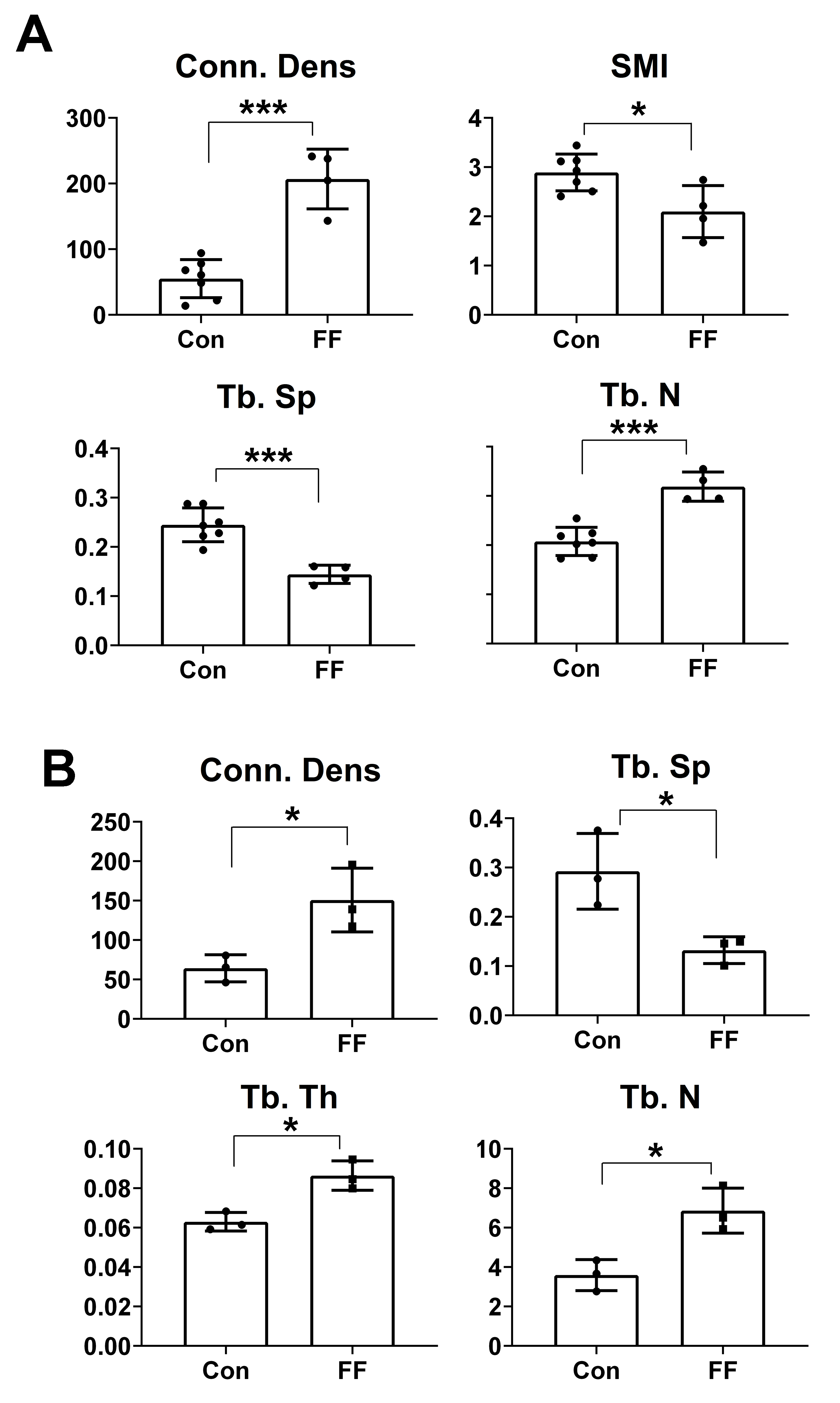

Supplement: S2 Fig — μCT quantitation of structural model index (SMI), trabecular number (Tb.N), trabecular thickness (Tb.Th), trabecular spacing (Tb.Sp) and connection density (Conn.Dens) of 3 month old control and FF. A) femur and B) vertebrae. Data are presented as mean ± SD. *p<0.05; ***p<0.001 as determined by unpaired t test. (TIF) [file pgen.1008244.s002.tif]

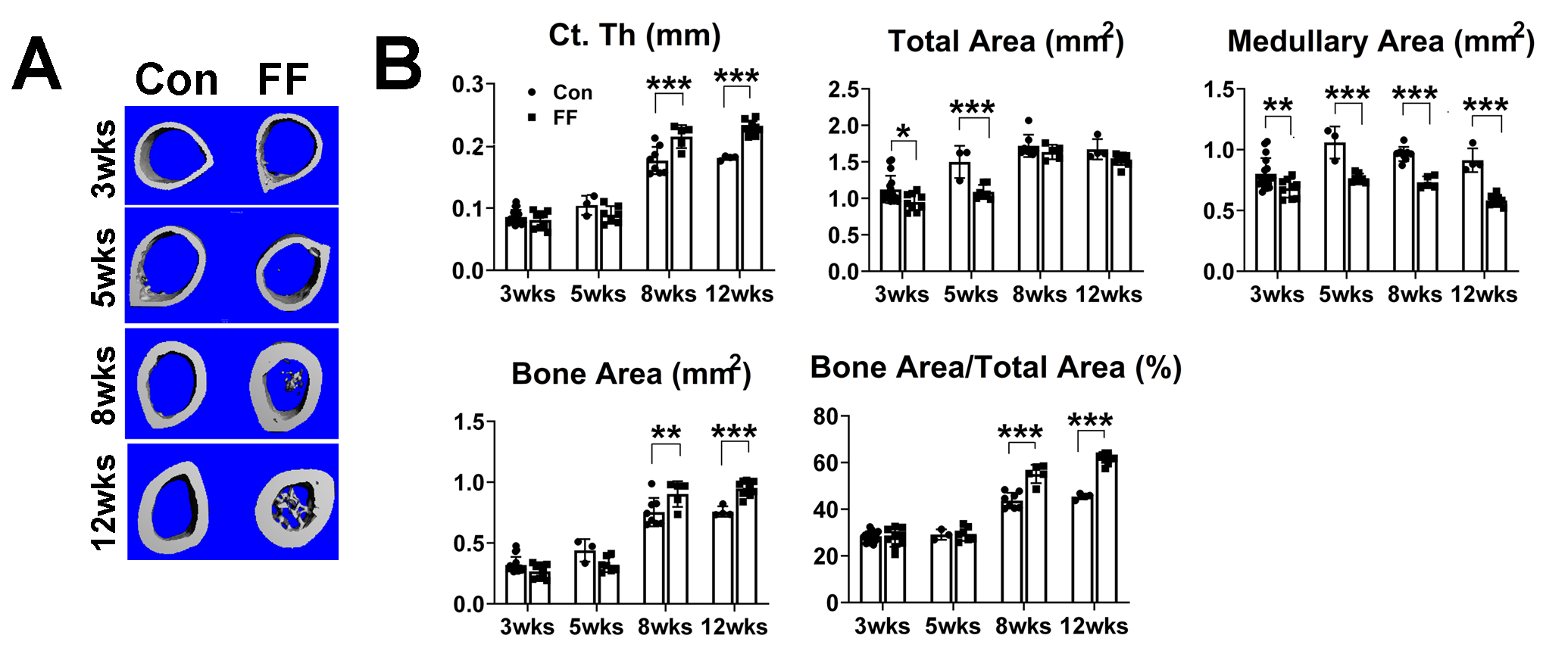

Supplement: S3 Fig — A) Age-dependent μCT images of femur diaphyseal mid-shaft region of FF and control littermates; B) μCT quantitation of A. Data are presented as mean ± SD. *p<0.05; **p<0.01; *** p<0.001 as determined by 2 way ANOVA with Holm-Sidak's post hoc analysis for multiple comparisons test. (TIF) [file pgen.1008244.s003.tif]

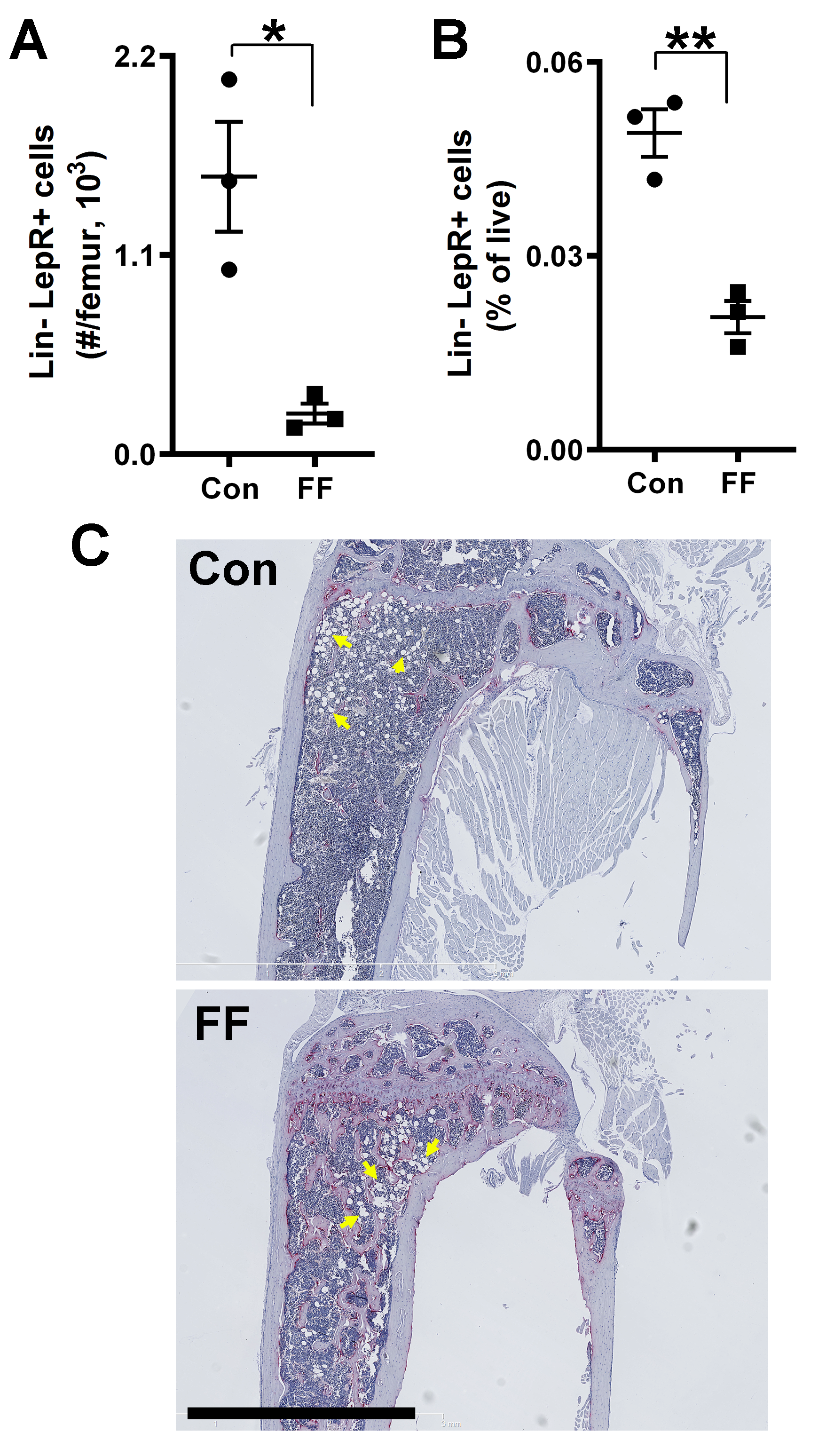

Supplement: S4 Fig — A) Flow cytometry assay of Lin-LepR+ cell number B) and ratio of Lin-Lep+ per total cell in femur marrow of 7 weeks old FF and control littermates. Data are presented as mean ± SD. *p<0.05; **p<0.01; as determined by unpaired t test. C) Histological section of 3 month old FF and control tibia stained for TRAP activity (red reaction product). Marrow adipocytes are present in both genotypes (arrow). (TIF) [file pgen.1008244.s004.tif]

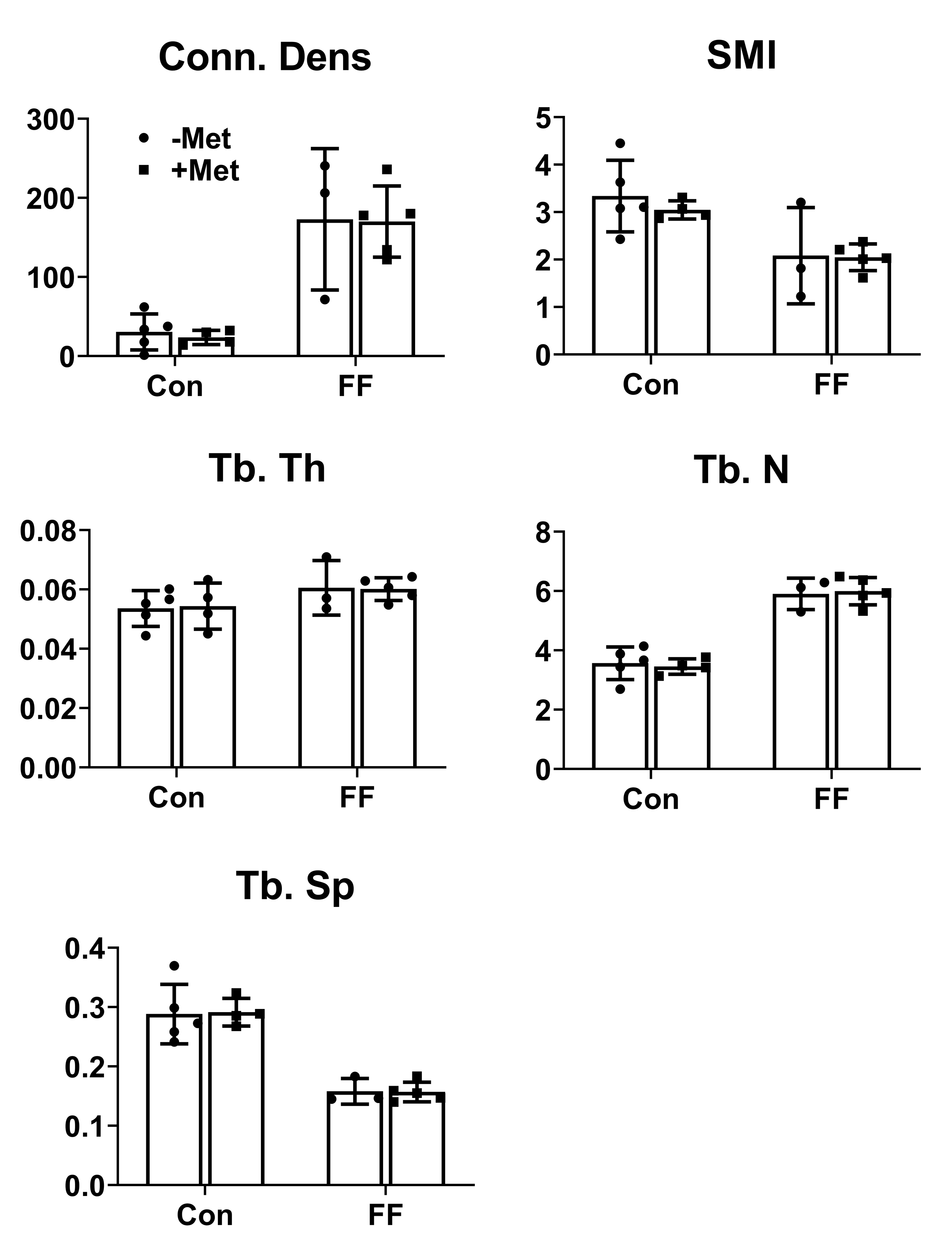

Supplement: S5 Fig — μCT quantitative analysis of distal femurs of FF mice following 3 months with or without metformin. Data are presented as mean ± SD. (TIF) [file pgen.1008244.s005.tif]

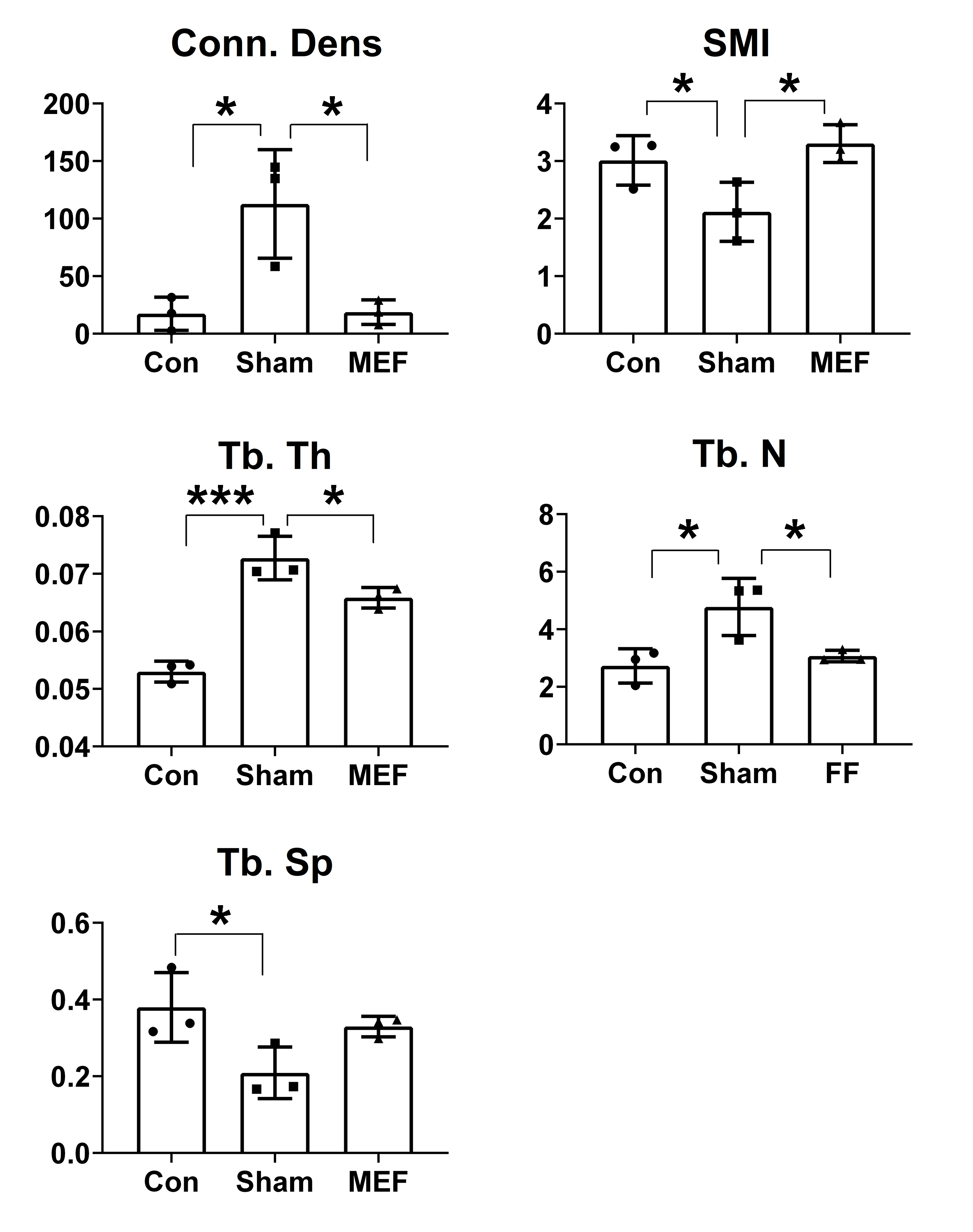

Supplement: S6 Fig — μCT quantitative analysis of distal femurs of FF mice 4 months after sham operation or MEF transplantation. Data are presented as mean ± SD. *p<0.05; *** p<0.001 as determined by ANOVA with Holm-Sidak's post hoc analysis for multiple comparisons test. (TIF) [file pgen.1008244.s006.tif]

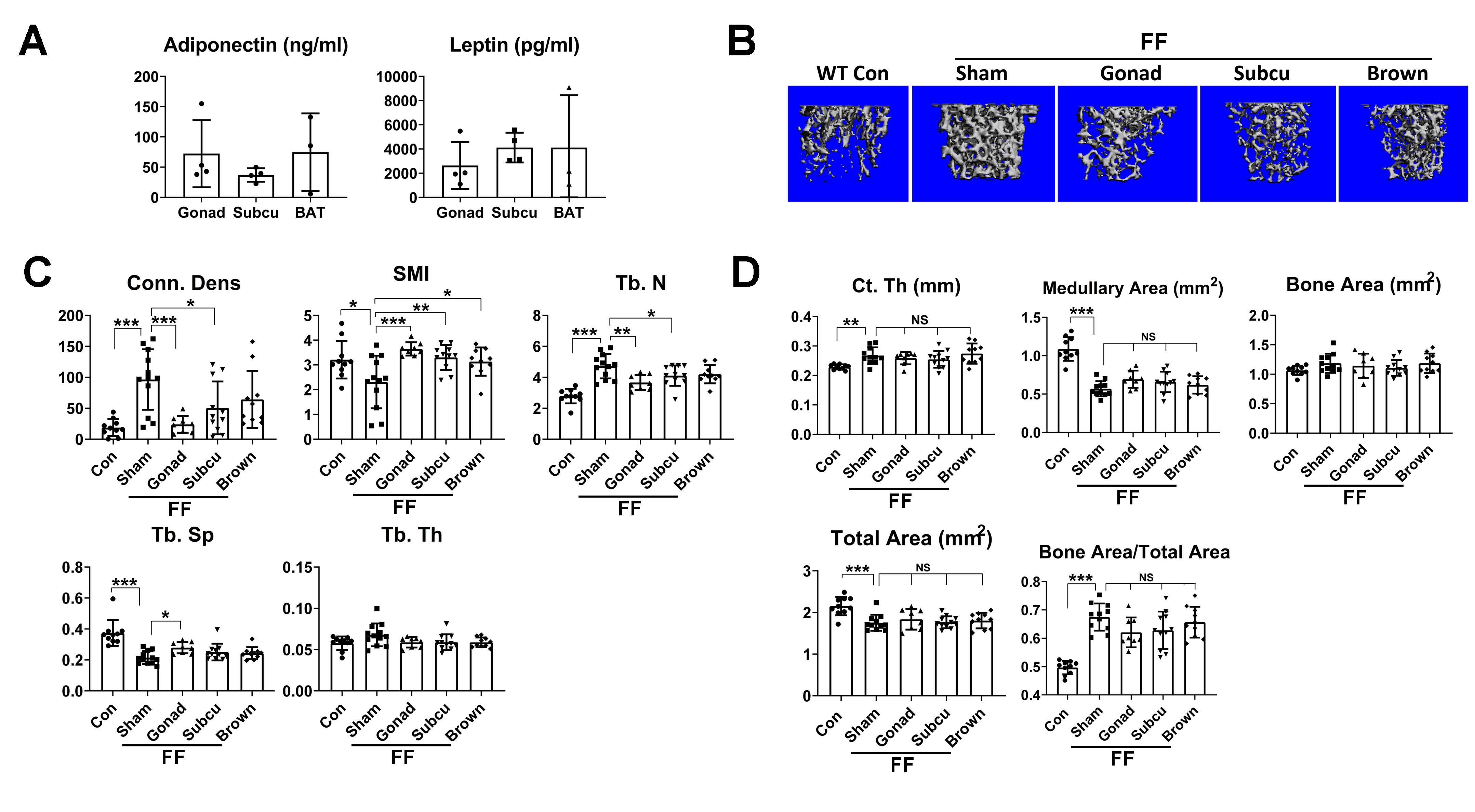

Supplement: S7 Fig — A) Serum leptin and adiponectin of FF mice 3 months after WT fat depot transplantation. μCT B) images and C) quantitative analysis of distal femurs of FF mice 3 months after sham operation or transplantation of various fat depots. D) μCT quantitative analysis of femur diaphyseal mid-shaft region of FF mice 3 months after sham operation or transplantation of various fat depots. Data are presented as mean ± SD. *p<0.05; **p<0.01; *** p<0.001; NS, not significant as determined by ANOVA with Holm-Sidak's post hoc analysis for multiple comparisons test. (TIF) [file pgen.1008244.s007.tif]

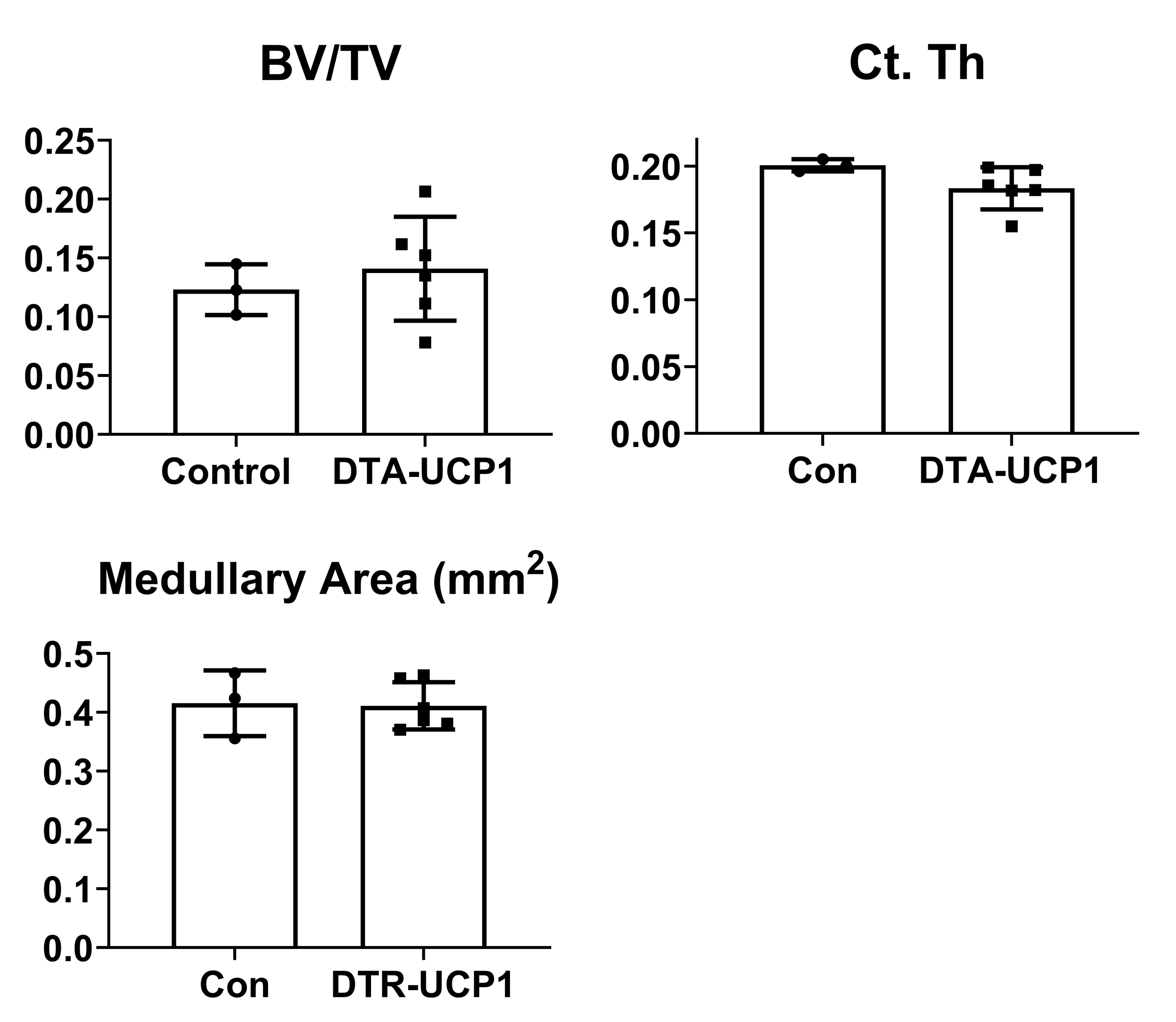

Supplement: S8 Fig — μCT quantitative analysis of femurs of three month old DTA-UCP1 Cre mice. Data are presented as mean ± SD. (TIF) [file pgen.1008244.s008.tif]

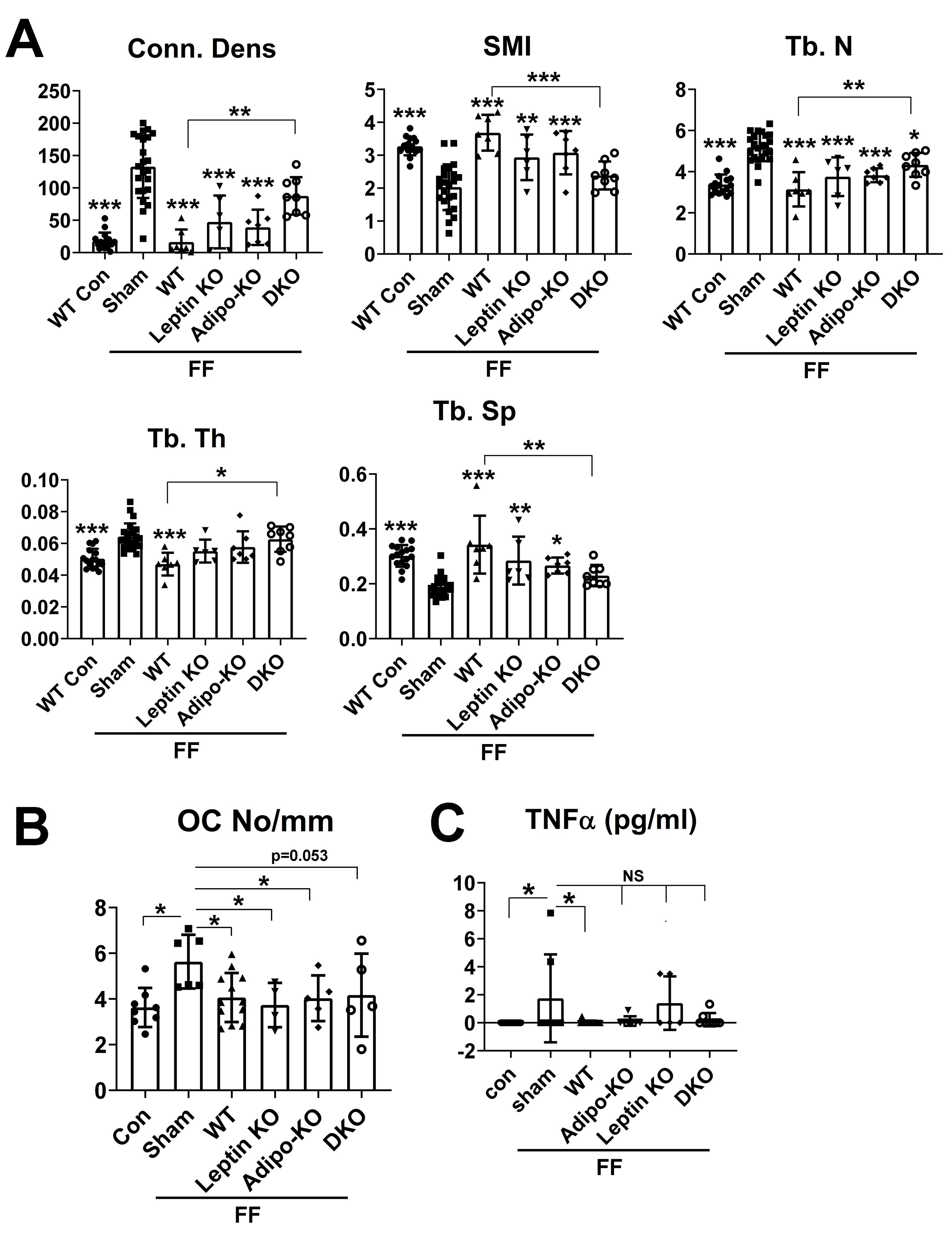

Supplement: S9 Fig — A) μCT analysis of distal femurs of FF mice 3 months after sham operation or transplantation of fat derived from WT or adipokine-deficient mice; B) Histomorphometric analysis of osteoclast number of FF and control femur 3 months after sham operation or transplantation of fat derived from WT or adipokine-deficient mice; C) Serum TNFα of control mice and FF mice 3 months after sham operation or transplantation of fat derived from WT or adipokine-deficient mice. Data are presented as mean ± SD. *p<0.05; **p<0.01; *** p<0.001 as determined by ANOVA with Holm-Sidak's post hoc analysis for multiple comparisons test. A) Comparison with Sham except where detailed. (TIF) [file pgen.1008244.s009.tif]
